# PHRONESIS: A One‐Shot Approach for Sequential Assignment of Protein Resonances by Ultrafast MAS Solid‐State NMR Spectroscopy

**DOI:** 10.1002/cphc.202200127

**Published:** 2022-05-19

**Authors:** Tata Gopinath, Veliparambil S. Manu, Daniel K. Weber, Gianluigi Veglia

**Affiliations:** ^1^ Department of Biochemistry, Molecular Biology & Biophysics University of Minnesota 321 Church St. SE Minneapolis MN 55455 USA; ^2^ Current address: NMRFAM Department of Biochemistry University of Wisconsin 433 Babcock Drive Madison WI 53706-1544 USA

**Keywords:** solid-State NMR, fast magic angle spinning, ^1^H detection, multi-acquisition, protein sequential assignment

## Abstract

Solid‐state NMR (ssNMR) spectroscopy has emerged as the method of choice to analyze the structural dynamics of fibrillar, membrane‐bound, and crystalline proteins that are recalcitrant to other structural techniques. Recently, ^1^H detection under fast magic angle spinning and multiple acquisition ssNMR techniques have propelled the structural analysis of complex biomacromolecules. However, data acquisition and resonance‐specific assignments remain a bottleneck for this technique. Here, we present a comprehensive multi‐acquisition experiment (PHRONESIS) that simultaneously generates up to ten 3D ^1^H‐detected ssNMR spectra. PHRONESIS utilizes broadband transfer and selective pulses to drive multiple independent polarization pathways. High selectivity excitation and de‐excitation of specific resonances were achieved by high‐fidelity selective pulses that were designed using a combination of an evolutionary algorithm and artificial intelligence. We demonstrated the power of this approach with microcrystalline U‐^13^C,^15^N GB1 protein, reaching 100 % of the resonance assignments using one data set of ten 3D experiments. The strategy outlined in this work opens up new avenues for implementing novel ^1^H‐detected multi‐acquisition ssNMR experiments to speed up and expand the application to larger biomolecular systems.

## Graphical abstract

**A comprehensive** solid‐state NMR experiment for sequential assignment of protein resonances – PHRONESIS – is presented.

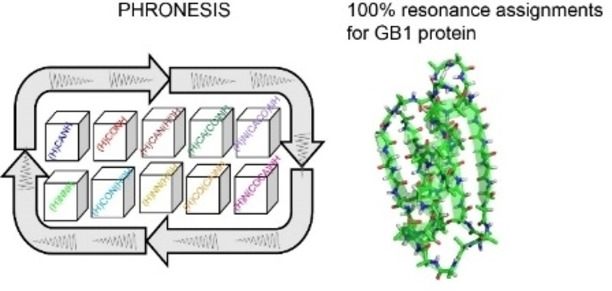

## Introduction

Solid‐state NMR (ssNMR) spectroscopy is a powerful technique for analyzing the structure and dynamics of insoluble, crystalline, and fibrillar biomacromolecules.[^1^, ^2^, ^3^, ^4^, ^5^, ^6^] The past few years have witnessed a significant effort to speed up the ssNMR experiments using ultrafast magic angle spinning (MAS), dynamic nuclear polarization (DNP), paramagnetic relaxation enhancement (PRE), ultra‐high‐field magnets, and ^1^H detected experiments.[^7^, ^8^, ^9^, ^10^, ^11^, ^12^, ^13^, ^14^, ^15^, ^16^, ^17^, ^18^] Additionally, the introduction of multi‐acquisition polarization optimized experiments (POE) has further boosted data acquisition[^19^, ^20^, ^21^, ^22^, ^23^] for both solution‐ and ssNMR spectroscopy.[^19^, ^20^, ^21^, ^22^, ^23^, ^24^, ^25^, ^26^, ^27^, ^28^, ^29^, ^30^, ^31^, ^32^] Indeed, ^1^H detection has significantly improved the sensitivity of fast and ultrafast MAS experiments for fully protonated protein samples; however, the broad line widths of the ^1^H resonances reduce the spectral resolution.[^8^, ^14^, ^15^, ^33^, ^34^] The latter is often exacerbated by conformational heterogeneity, restricting the range of applications of ssNMR spectroscopy.[^35^, ^36^, ^37^] Nonetheless, higher dimensionality, multiple acquisitions, and multiple receiver ssNMR experiments promise to overcome these challenges.^[60]^

The first step to obtaining atomic resolution information of biomacromolecules is the resonance‐specific assignment. A standard approach for unambiguous resonance assignments is to acquire several redundant 3D data sets and correlate the spin systems (residues) via multiple pathways.[^6^, ^14^] In principle, simultaneous mapping of the entire protein backbone can be achieved using broadband polarization transfer schemes, a unique feature of ssNMR made possible by the strong dipolar coupling network.[^38^, ^39^] However, most of the current 3D ssNMR experiments, including multi‐acquisition methods, utilize either CA‐ or CO‐based polarization transfer schemes[^1^, ^2^, ^3^, ^4^] due to the difficulties associated with spectral selectivity of multiple pathways. Another drawback is the need to sample the full ^13^C spectrum, leading to an exponential increase of the acquisition time for multidimensional experiments. Ideally, the deconvolution of specific polarization pathways would help decoding nuclear spin connectivity for residue‐specific assignments. Nevertheless, the fidelity of the current selective pulses prevented the quantitative transfer of selective polarization.^[40]^ To overcome these challenges, we designed new selective pulses using GENETICS‐AI (Generator of Triply Compensated Pulses‐Artificial Intelligence) software[^42^, ^43^] and combined them with broadband polarization transfers into a novel comprehensive 3D experiment PHRONESIS (Proton detected orpHan spin polaRization for prOteiN sequEntial asSIgnment using SsNMR). PHRONESIS utilizes CA, CO, and ^15^N pathways to acquire ten 3D experiments simultaneously. PHRONESIS correlates the N(i)‐H(i) spin pairs through CA(i‐1), CA(i), CO(i‐1), CO(i), HA(i), N(i‐1), and N(i+1) chemical shifts, providing a one‐shot approach for unambiguous assignments of proteins resonances.

## Results and Discussion

### Design and Optimization of the Building Blocks of PHRONESIS

**Figure 1:**
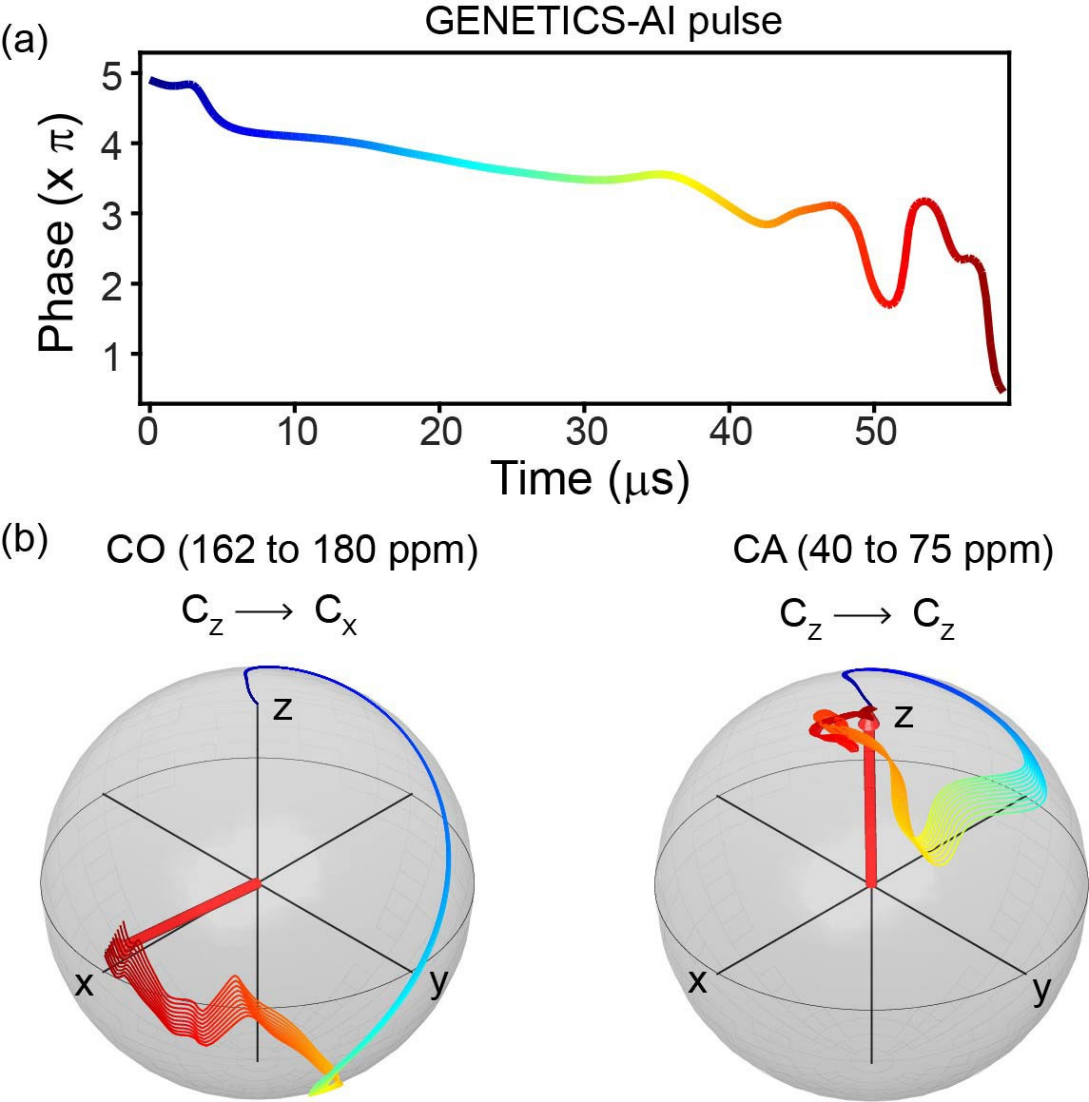
(a) GENETICS‐AI pulse for CO excitation obtained from phase modulation and constant RF amplitude (16.7 kHz) with a total length of 59 μs. (b) During the pulse, evolution of CO and CA magnetization trajectories are projected on the Bloch spheres for the CO excitation and CA refocusing (Figures S1 and S2).

PHRONESIS consists of sequential building blocks with multi‐directional broadband transfer pathways that are encoded with robust selective pulses (Supplementary Information, section S1).^[43]^ The selective excitation was achieved using high‐fidelity ^13^C selective pulses designed *ad hoc* using an evolutionary algorithm combined with artificial intelligence (AI).^[43]^ Unlike conventional selective shaped pulses, the RF amplitude is constant, and the spectral selectivity is obtained exclusively by phase modulation.^[43]^ These pulses have a fidelity greater than 0.99 and are compensated for offset‐dependent evolution during the pulse execution, avoiding spectral distortions typical for conventional selective shaped pulses.^[40]^ Additionally, the pulse duration is significantly shorter, making the GENETICS‐AI pulses less prone to signal losses via T_2_ relaxation mechanism. The high‐fidelity operation of these selective pulses is shown in Figure 1, where spin evolution trajectories for ten different chemical shift values CO and CA spectral regions are represented during the CO excitation pulse. At the end of the pulse, the evolution of the polarization from the CA spectral region is completely refocused. For example, we applied these selective pulses to ^13^C‐spectra of microcrystalline GB1 (Figure S1), with the simulated spectral profiles represented in Figure S2. The CA and CO spectra obtained from cross‐polarization (CP) followed by the selective pulses show no signal losses relative to the reference ^13^C CP spectrum. In contrast, either Gaussian or Q5 cascade selective pulses result in approximately 10 to 40 % signal loss (Figure S1c,d).[^40^, ^44^]

Before building the complete experiment, we optimized each block necessary to create multiple spin pathways, *i. e*., the simultaneous CP (SIM‐CP), SPECIFIC‐CP, and DREAM sequences (Supplementary Information, sections S2–S4).[^19^, ^45^, ^46^] In most multidimensional experiments, the RF matching conditions and phase cycling are optimized to avoid broadband polarization transfer, which would lead to spectral artifacts from undesired polarization pathways. Indeed, these orphan spin pathways can be recovered using selective GENETICS‐AI pulses. To achieve broadband polarization transfer from the ^1^H spin bath to CA, CO and ^15^N, the SIM‐CP sequence was applied with the ^13^C offset centered between the CA and CO regions, and the RF field amplitudes were matched to the ZQ (zero quantum) Hartmann‐Hahn condition (Supplementary Information, section S2). A comparison of the signal intensities of ^1^H detected 1D experiments with broadband SIM‐CP and CP is shown in Figures S3 and S4. The CA‐edited HCANH spectra obtained from SIM‐CP are similar to CP (Figure S3a). In contrast, the intensities of CO and ^15^N‐edited ^1^H spectra obtained from SIM‐CP are 74 % and 70 %, respectively, relative to the signal obtained from CP (Figures S3b,c, and S8). Note that the ^1^H density surrounding CO and ^15^N atoms is lower than CA and side‐chain carbons, leading to a loss of polarization under SIM‐CP conditions.^[19]^

The performance of the broadband SPECIFIC‐CP is demonstrated in Supporting Information section S3 and Figures S5d and S6d. The SPECIFIC‐CP sequence was implemented with a ^13^C spin‐lock pulse applied at 115 ppm for simultaneous polarization transfer between CA−N and CO−N spin pairs. The^15^N RF was optimized to satisfy the DQ (double quantum) recoupling conditions. Previous multi‐acquisition ssNMR experiments utilized two to four pathways originating from SPECIFIC‐CP or other NC transfer variants.[^19^, ^20^, ^21^, ^22^, ^23^, ^24^, ^25^, ^26^, ^27^, ^28^, ^29^, ^30^, ^31^, ^32^] In contrast, the broadband SPECIFIC‐CP sequence creates seven different pathways (Figure S8b). The intensities of the CANtr, CONtr, NCAtr, and NCOtr pathways, were 103 %, 72 %, 55 %, and 100 %, respectively, relative to CA and CO SPECIFIC‐CP transfers (Figures S5‐S6). The intensities of the remaining three residual pathways, CAres, COres, and Nres, were 40 %, 37 %, and 22 % (Figures S5–S6). Next, we optimized the homonuclear CC transfer for connecting the other polarization pathways. The DREAM recoupling sequence under fast MAS significantly improves the CC transfer efficiencies. Figure S7 shows the optimization of bidirectional (CACO and COCA) CC transfer, facilitated by broad matching conditions of DREAM recoupling (Supporting Information, section S4).

### Implementation of PHRONESIS

**Figure 2:**
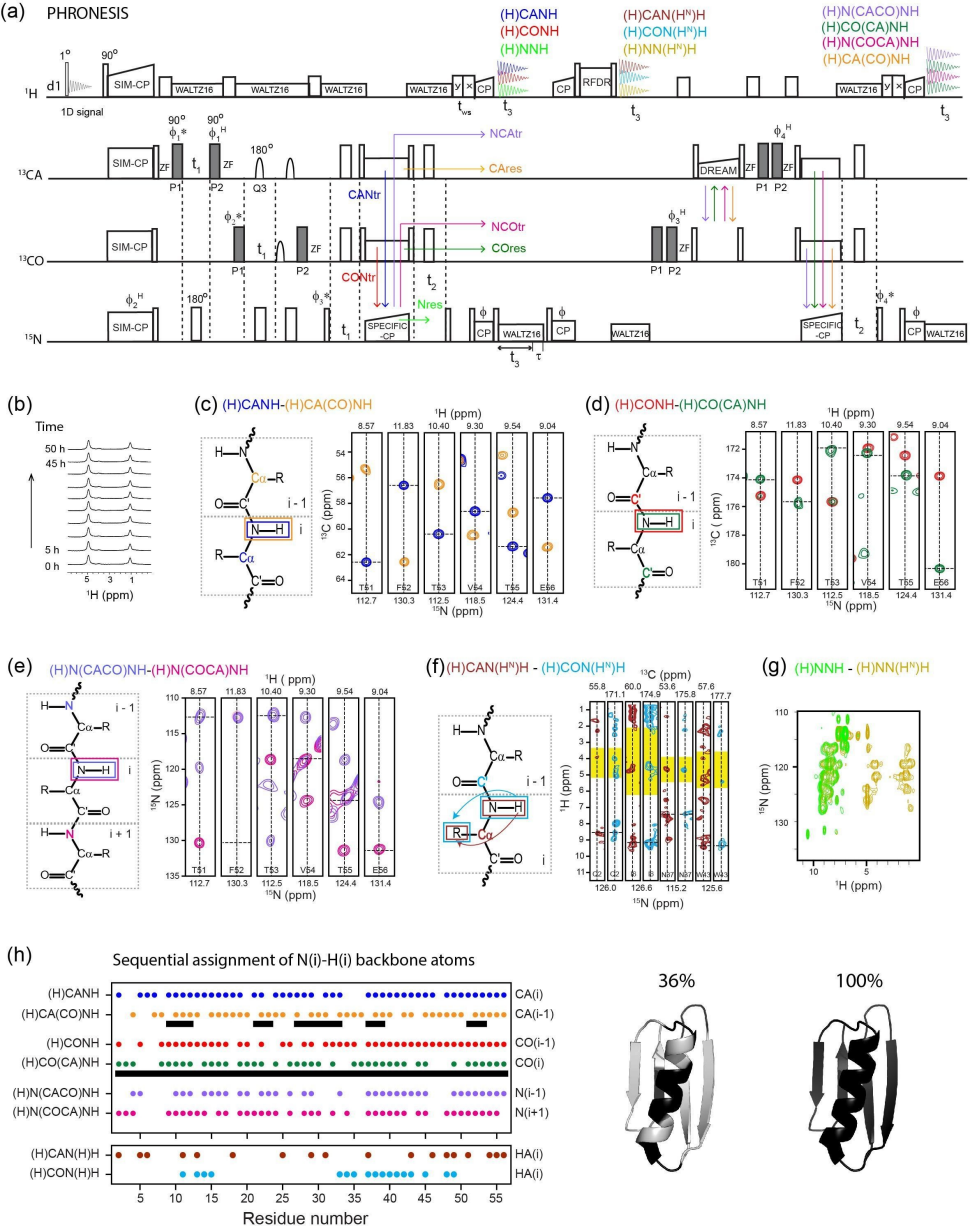
(a) PHRONESIS experiment for simultaneous acquisition of ten 3D spectra using ^1^H detected ultrafast MAS. The GENETICS‐AI pulses (gray rectangles) are applied on CA and CO to encode multiple pathways. (c–h) Sequential assignment of GB1 protein using ten 3D spectra (Table S1) acquired with 65 kHz MAS. (h) Summary of unambiguously resolved 3D cross peaks picked from each experiment. Cross peaks obtained from only the (H)CANH‐(H)CA(CO)NH pair yielded correct automatic assignments for only 36 % of NH spin systems, while 100 % accuracy was achieved if CO(i‐1) and CO(i) correlations were also included.

Once each building block was optimized, we assembled the entire PHRONESIS pulse sequence (Figure 2A). This experiment consists of four ^1^H acquisition periods. The 1^st^ acquisition records the water signal using a small flip angle pulse (1°). The resulting 1D spectra are used to monitor sample hydration and correct for possible drifting of the static magnetic field (B_**o**_) during the experiment. The 2^nd^, 3^rd,^ and 4^th^ acquisitions record four pairs of intra‐ and inter‐residue correlation data sets, (H)CANH‐(H)CA(CO)NH, (H)CONH‐(H)CO(CA)NH, (H)N(COCA)NH‐(H)N(CACO)NH, and (H)CAN(H^N^)H‐(H)CON(H^N^)H, and two pseudo‐3D spectra (H)NNH and (H)NN(H)H. First, the SIM‐CP period simultaneously transfers the polarization from the ^1^H spin bath to CA, CO, and ^15^N nuclei. The ^15^N, CA, and CO chemical shifts are co‐evolved in separate t_1_ periods using 90° excitation and flip‐back pulses (Figure 2a). The gray rectangles represent the GENETICS‐AI selective CA and CO pulses. After the flip‐back pulses, a z‐filter is applied to retrieve only z‐polarization and remove any spectral artifacts associated with transverse signals. Thanks to the GENETICS‐AI pulses, the CA and CO polarization pathways are treated as separate channels. Note that the ^13^C hard pulses represented on CA and CO correspond to a single pulse applied at 100 ppm (Table S1). During t_1_, to refocus the CO‐CA J‐couplings, 180° Q3 pulses were applied. After t_1_ evolution, the broadband SPECIFIC‐CP creates four transferred polarization pathways (CANtr, CONtr, NCAtr, and NCOtr) and three residual polarization pathways (CAres, COres, and Nres). The four ^13^C polarization pathways (CAres, COres, NCAtr, and NCOtr) are stored along the z‐direction. Whereas the ^15^N polarization (CANtr and CONtr, and Nres) is evolved during t_2_ followed by a NH CP to record (H)CANH, (H)CONH, and (H)NNH 3D spectra in the 2nd acquisition period. After NH CP, ^15^N residual polarization along the z‐direction is recovered by incorporating an additional decoupling delay (τ in Figure 2a).^[21]^ The residual ^15^N polarization is then transferred to H^N^ followed by short HH RFDR mixing, which gives (H)CAN(H^N^)H, (H)CON(HN^N^)H, and NN(H^N^)H spectra in the 3^rd^ acquisition. Next, the four ^13^C polarization pathways (CAres, COres, NCAtr, and NCOtr) stored along the z‐direction are flipped into the transverse plane by a 90° pulse followed by a bidirectional DREAM (CACO and COCA transfer) and SPECIFIC‐CP (CAN and CON transfer). Under DREAM and SPECIFIC‐CP, residual CA and CO polarization (CAres and COres) follows CACON and COCAN pathways, respectively, wherease NCAtr and NCOtr polarization follows NCACON and NCOCAN pathways, respectively. Two pairs of GENETICS‐AI pulses on CA and CO were applied before and after the DREAM to phase‐encode the four pathways. The resulting ^15^N polarization from these four pathways (CACON, COCAN, NCACON, and NCOCAN) is evolved during a t_2_ period followed by a NH CP and the 4^th^ acquisition, which gives four 3D spectra (H)CA(CO)NH, (H)CO(CA)NH, (H)N(CACO)NH, and (H)N(COCA)NH. The 3D PHRONESIS data sets are acquired with a unique Hadamard phase encoding procedure obtained by switching the phases of four pulses, ϕ_1_^H^, ϕ_2_^H^, ϕ_3_^H^, and ϕ_4_^H^ (Figure 2a), between x and −x.[^22^, ^47^, ^48^, ^49^] The phase encoding of ten polarization pathways is represented by the Hadamard matrix columns (Figures S9 and S19). The decoding of multiple 3D spectra is obtained by a linear combination of the data sets according to respective columns of the Hadamard matrix. Figure S19 describes the PHRONESIS data processing protocol, along with pulse sequence and processing scripts. Each decoding process resembles a phase cycle and, therefore, decodes a single coherence transfer pathway while eliminating unwanted signals.

### Complete Resonance Assignment of U‐^13^C,^15^N Microcrystalline GB1

Figure 2b–h shows the sequential assignment of N, CA, CO, HA, and H^N^ atoms of uniformly ^13^C,^15^N labeled GB1 protein using ten 3D PHRONESIS spectra. The total experimental time was 52 h, with a net time saving of ∼50 % relative to conventional 3D experiments (∼103 h, see Experimental Section). All acquisition parameters are reported in Table S1. As shown in Figure 2h, the 3D spectra unambiguously resolved between 60 to 80 % of the resonances. Due to several breaking points for the CA(i) and CA(i−1) connections in the (H)CANH‐(H)CA(CO)NH spectral pair, only 36 % of NH spin systems were assigned correctly using the semi‐automatic algorithm of the I‐PINE server.^[50]^ However, the backbone sequential connectivities dramatically improved once the CO(i−1) and CO(i) correlations were included using the (H)CONH and (H)CO(CA)NH datasets, reaching an accuracy of 100 % (Table S2). These assignments were further confirmed by (H)N(CACO)NH and (H)N(COCA)NH data sets. Therefore, simultaneous mapping of multiple spin pathways offered by PHRONESIS provides sufficient data for a complete and robust sequential backbone assignment. While CA and CO correlations were enough for GB1, the inclusion of N(i−1) and N(i+1) connectivities from the (H)N(CACO)NH and (H)N(COCA)NH data sets will be critical to assign larger proteins. Although this pathway is not implemented into commonly used automated assignment software, it can be included to manually resolve possible assignment ambiguities. After the assignment of the backbone H^N^, N, CA, and CO resonances, it is possible to obtain intra‐residue H^N^‐HA cross‐peaks from the (H)CAN(H^N^)H, (H)CON(H^N^)H, (H)NNH, and (H)NN(H^N^)H data sets. Note that to optimize the intra‐residue H^N^‐HA transfer, we used a short RFDR mixing period (∼738 μs) before the 3^rd^ acquisition. Since the HH‐RFDR mixing results in a non‐selective transfer, it can lead to long‐range correlations even at short mixing times, which may complicate the spectral analysis. Therefore, we analyzed the data by comparing the (H)CAN(H^N^)H and (H)CON(H^N^)H strip plots with typical residue‐specific HA (shaded in yellow) and HB (pink) spectral ranges according to the BMRB database (Figure S18). From this analysis, we obtained HA assignments for 30 residues. Finally, a prominent feature of PHRONESIS is the ability to monitor the signal of water throughout the entire experiment. Figures 2a and S11a show the 1D water spectra of PHRONESIS. This feature enables one to monitor both the magnetic field drift and sample hydration conditions. In fact, most of the ssNMR probes do not possess a lock channel, and the RF heating can also cause severe loss of water from biological samples. While little change was observed over the short experimental time required for GB1, more significant changes are expected for lower sensitivity samples with longer acquisition times. The water reference spectra can be included in SPARKY′s routine in the data processing to compensate for possible frequency drifts. Although we demonstrated the application of PHRONESIS for crystalline proteins, we envision the application of this method to more challenging biological systems such as fibrillar and membrane‐bound proteins. For homogenous systems with relatively narrow linewidths, e. g., some fibrillar proteins, acquiring multiple spectra via PHRONESIS will be straightforward with significant time‐saving. However, for heterogeneous systems such as membrane proteins reconstituted in fully hydrated lipid bilayers, the application of PHRONESIS will be more challenging as the resolution of ^1^H‐detected dimensions will be limited by the broadening of the ^1^H resonances caused by intrinsic conformational dynamics and sample heterogeneity (i. e., static disorder).^[51]^

## Conclusions

In conclusion, we presented a comprehensive ssNMR experiment, PHRONESIS, that generated ten 3D data sets for the sequential assignment of proteins using ^1^H‐detection under ultrafast MAS. PHRONESIS combines broadband polarization transfer schemes, high‐fidelity selective pulses, and Hadamard‐encoding techniques to resolve overlapping spin systems in multiple dimensions. Beyond the sheer interest of time‐saving, simultaneous mapping of the entire protein backbone using PHRONESIS ensures reliable chemical shift measurements, minimizing spectral misalignments and peak picking ambiguities. PHRONESIS is a blank canvas for designing novel multidimensional ssNMR experiments by concatenating different multi‐dimensional pulse sequences, speeding up the spectral assignment, and structure determination of complex macromolecules.[^19^, ^20^, ^21^, ^22^, ^23^, ^24^, ^25^, ^26^, ^27^, ^28^, ^29^, ^30^, ^31^, ^32^]

## Experimental Section

### Protein Preparation and NMR Spectroscopy

U‐^13^C,^15^N labeled microcrystalline GB1 (β1 immunoglobulin binding domain of protein G, crystal form A) protein sample was prepared using the protocol described previously.^[52]^ Approximately 2 mg of GB1 microcrystals in residual precipitant solution were packed into a 1.3 mm rotor. All the solid‐state NMR experiments were implemented on a Bruker 600 MHz spectrometer equipped with a 1.3 mm fast MAS probe and Avance NEO console using the TOPSPIN 4.0.9 software.

All the spectra were acquired with a MAS rate of 65 kHz. A sample temperature of 25 °C was maintained by setting the RF coil temperature to −18 °C to compensate for the heat induced by fast spinning as measured from the water resonance frequency. The 3D PHRONESIS pulse sequence (Figures 2a) was implemented with a two‐step phase cycle of ϕ pulse, and sixteen‐step Hadamard phase encoding (ϕ_1_^H^, ϕ_2_^H^, ϕ_3_^H^, ϕ_4_^H^), which corresponds to 32 scans. Pulse program and processing scripts are given at the end of Figure S19. Heteronuclear and homonuclear polarization transfer is obtained by using either ZQ (zero quantum) or DQ (double quantum) recoupling conditions (Table S1).[^45^, ^46^, ^53^, ^54^] After the flip back pulse, a z‐filter (2 ms) was applied. To speed up the transverse signal dephasing during z‐filter, we applied ^1^H RF with HORROR recoupling condition (ν_1H_=ν_r_/2 or ν_r_). Broadband SIM‐CP and SPECIFIC‐CP were optimized with ^13^C spin‐lock pulses applied at the center of CA and CO spectral regions (115 to 125 ppm). The t_1_ and t_2_ dimensions were acquired in States‐TPPI mode.^[40]^ During t_1_ evolution, the offset switching to CA and CO is obtained by t_1_ dependent phase modulation of ϕ_1_* and ϕ_2_* pulses, respectively (Figure 2a). For GB1, the CA spectral width is approximately twice that of CO and ^15^N. Therefore, to obtain parallel t_1_ evolution, CA was evolved with 225 μs dwell time and 36 increments. For CO and ^15^N, two sets of t_1_ data sets were acquired with 18 increments, 450 μs dwell time,^[19]^ corresponding to a total t_1_ evolution of 7.88 ms for CA and 7.65 ms for CO and ^15^N. Two identical t_1_ data sets for CO and ^15^N edited spectra were added during the processing (Figure S19). For all the 3D experiments, the ^15^N chemical shift was evolved during t2 with 450μs dwell time and 18 increments corresponding to a maximum t_2_ evolution time of 7.65 ms (Table S1). During CO t_1_ evolution, the J‐coupling between CA and CO is refocused by applying a 180° Q3 cascade selective pulses. ^1^H detected 1D experiments were used for optimizing the RF parameters (Figures S3–S7). ^13^C detection was used for calibrating the selective pulses (Figure S1). Optimal RF values were calibrated by recording an array of 1D spectra using “popt” (parameter optimization) experimental setup available in Bruker TOPSPIN. The 1D pulse sequences of Figures S3–S7 can be combined into single 1D pulse sequences with multiple acquisitions. However, the current “popt” protocol cannot be implemented with multi‐acquisition periods. Therefore, all the RF parameters (Table S1) were optimized using single acquisition 1D pulse sequences.

To compare the relative efficiencies of PHRONESIS relative to conventional single acquisition 3D experiments,^[55]^ we measured the intensities of 1D spectra (Figure S10) obtained from the first increment (*i. e*., t_1_=t_2_=0). The intensity of the PHRONESIS (H)CANH 1D spectrum is 97 % or a factor of 0.97 relative to the conventional experiment because the transfer efficiencies of CA SIM‐CP and CAN SPECIFIC‐CP match (*i. e*., ∼100 %) with CP and SPECIFIC‐CP (Figures S3a, S5d, and S8). The intensities of (H)N(COCA)NH and (H)CONH are respectively 75 % and 51 %, due to signal loss during SIM‐CP and SPECIFIC‐CP (Figure S3b,c, and S5d, S6d). Similarly, the intensities of (H)CO(CA)NH, (H)CA(CO)NH, and (H)N(CACO)NH are respectively 25 %, 30 %, and 20 %, which are consistent with polarization transfer efficiencies shown in Figures S3–S8. The HH cross‐peak spectra acquired in the 3^rd^ acquisition (Figure 2), (H)CAN(H^N^)H, (H)CON(H^N^)H, and (H)NN(H^N^)H, utilize weak ^15^N residual polarization from NH reverse‐CP,^[21]^ and therefore the corresponding intensities are 6 to 10 % relative to single acquisition pulse sequences. The conventional 3D experiments use single t_3_ acquisition, water suppression, and t_1_ and t_2_ evolution periods. In contrast, PHRONESIS uses four t_3_ acquisition periods, three t_1_ and two t_2_ evolution periods, and two water suppression periods with 100 and 25 ms duration. However, these additional pulse sequence delays (∼70 ms) of PHRONESIS only increase the experimental time by ∼3 % since the major contribution to experimental time is the recycle delay (2 s). To estimate the required experimental time for each single acquisition 3D experiment, we used the formula T_exp_^conventional^=k^2^*[T_exp_^PHRONESIS^]*0.97. Here ‘k‘ is the percentage factor (k=x/100 for x %) of 1D PHRONESIS signal intensities relative to single‐acquisition experiments (Figure S10), and the factor 0.97 accounts for the 3 % increase of PHRONESIS experimental time due to additional delays. For example, for single acquisition 3D (H)N(COCA)NH, to achieve similar S/N and resolution, the required experimental time is 28.4 h (=0.75^2^*52*0.97 h) with k=0.75 (or 75 %, Figure S10) and T_exp_^PHRONESIS^=52 h (Figure 2, Table S1). This approximation assumes linear adjustment of the number of scans for conventional 3D experiments for achieving similar S/N. From this analysis, the calculated total experimental time for conventional 3D experiments is approximately 103 h or a 100 % increase relative to the PHRONESIS experimental time (52 h). Note that the net time saving from low sensitive data, (H)CAN(H^N^)H and (H)CON(H^N^)H of 3rd acquisition, is insignificant. It is generally not feasible to optimize the experimental time (or the number of scans) for low and high‐sensitive experiments that are acquired simultaneously. However, the multi‐acquisition architecture of PHRONESIS and the Hadamard encoding scheme give other possibilities to optimize the total acquisition time by incorporating other experiments. For example, the signals of 2^nd^ acquisition are relatively intense, and these polarization pathways are independent of 3^rd^ and 4^th^ acquisition experiments (Figure 2a, S10). Therefore, as shown from the 1D spectra of Figure S14, PHRONESIS can be modified by incorporating single or multiple RFDR mixing periods^[56]^ before the 2^nd^ acquisition to obtain high‐intensity HH cross‐peaks. Similarly, it is also possible to probe the protein dynamics by incorporating ^15^N T1ρ spin lock periods^[6]^ before (H)CANH and (H)CONH (2nd acquisition of Figure 2a) without impacting the ^13^C stored polarization that is acquired in the 4th acquisition. In other words, PHRONESIS can be modified to include several higher dimensional experiments.

### Spectral Processing and Sequential Assignment

The PHRONESIS data from four acquisition periods are first divided into four files using the “split“ command available in TOPSPIN and then processed using NMRPipe scripts (Figure S19).[^57^, ^58^] Each acquisition consists of sixteen interleaved data sets obtained by Hadamard phase cycling of four pulses {ϕ_1_^H^, ϕ_2_^H^, ϕ_3_^H^, ϕ_4_^H^}=(x,x,x,x); (x, x, x, −x); ……..; (x,−x,−x,x).[^22^, ^49^] The Hadamard matrix columns (Figure S9) represent the signs of ten 3D polarization pathways in sixteen interleaved scans. As shown in Figure 2a, (H)CONH (2nd acquisition), (H)CON(H^N^)H (3rd acquisition) pathways are not affected by ϕ_1_^H^, ϕ_2_^H^, ϕ_3_^H^, ϕ_4_^H^ phases. Therefore, these data sets are obtained by adding all sixteen interleaved scans (Figure S9). The CA edited data sets (H)CANH, (H)CAN(H^N^)H, and (H)CA(CO)NH are only affected by the ϕ_1_^H^ phase; therefore, these data sets are obtained by the linear combination [1−1+1−1+1−1+1−1+1−1+1−1+1−1+1−1]. Similarly, other 3D data sets are deconvoluted according to the Hadamard matrix columns (Figure S9). For each 3D processing, the decoding script is incorporated at the beginning of the NMRPipe processing file. (H)CAN(H^N^)H and (H)CON(H^N^)H (3^rd^ acquisition) are the least sensitive because of the weak ^15^N residual polarization (∼10 %) from NH reverse‐CP. Therefore, the corresponding 3D data was processed with 100 Hz line broadening in the direct dimension. All the remaining eight 3D data sets were processed with a 90° shifted sine bell window function. For all the data sets, t_1_ and t_2_ indirect dimensions were processed without any phase correction, and only zero‐order phase correction was applied in the direct t_3_ dimension. The 3D spectra (H)NNH and (H)NN(H)H have the same ^15^N chemical shift evolution in t_1_ and t_2_. Therefore, the data were processed as a 2D by summing the F1 planes (Figure 1g). The six 3D spectra, (H)CANH, (H)CA(CO)NH, (H)CONH, (H)CO(CA)NH, (H)N(CACO)NH, and (H)N(COCA)NH, were manually analyzed using NMRFAM‐SPARKY(Figures S15–S18).^[59]^ The resonances were assigned to ^1^H‐^15^N spin systems using the following criteria: 1) they were picked by the peak‐picking function of NMRFAM‐SPARKY; 2) they were resolved from the cross‐peaks of other residues; and 3) each dimension matched within a tolerance of 50 % of the average FWHM (i. e., 150 Hz/0.25 ppm for HN, 75 Hz/0.45 ppm for CA, 60 Hz/0.4 ppm for CO, and 57.5 Hz/1.9 ppm for ^15^N). The data were then uploaded into the I‐PINE web server for automatic assignment.^[50]^ Due to several breaking points, only 36 % of NH spin systems were assigned correctly using (H)CANH‐(H)CA(CO)NH spectral pair. When the (H)CONH and (H)CO(CA)NH data were included, unambiguous N−H assignments were obtained with an accuracy of 100 % (Table S2). The correctness of the resonance assignments was confirmed by comparison to previously published assignments.^[9]^ The backbone chemical shifts (N, CA, CO, H^N^) were then overlaid on (H)CAN(H^N^)H, (H)CON(H^N^)H, and (H)NN(H^N^)H data sets for assigning intra‐residue HA chemical shifts (Figure S18). Note that the ^1^H dimension (t3) consists of H^N^, HA, and side‐chain chemical shifts. Only the resolved H^N^ peaks of (H)CAN(H^N^)H, (H)CON(H^N^)H, and (H)NN(H^N^)H were analyzed to obtain the intra‐residue HA shifts. Side‐chain ^1^H chemical shifts were not analyzed due to low signal intensities and spectral overlap. The intensity of (H)NN(H^N^)H is higher than (H)CAN(H^N^)H, (H)CON(H^N^)H (Figure S10). However, due to identical F1 and F2 dimensions and low ^1^H resolution, (H)NN(H^N^)H did not provide any new assignments. Note that even at a short HH RFDR mixing time (0.7 ms), the H^N^ polarization leads to long‐range correlations. Therefore, to guide the assignment, the expected HA peak ranges were taken (shaded in Figures 2f and S18) from BMRB (Biological Magnetic Resonance Data Bank) database. We assigned the most intense peak in the HA shaded region with a signal‐to‐noise ratio greater than 4. Depending on the resolution in the NCA and NCO planes, HA peaks are resolved in either (H)CAN(H^N^)H or (H)CON(H^N^)H or both data sets. Using this analysis, we assigned 18 HA peaks from (H)CAN(H^N^)H, and 17 HA peaks from (H)CON(H^N^)H, where 5 HA peaks were identified in both data sets. An additional 15 H^N^ peaks are resolved in (H)CAN(H^N^)H and (H)CON(H^N^)H, but the corresponding HA peaks are not detected due to low signal intensities.

## Notes

The authors declare no competing financial interest.

## Conflict of interest

The authors declare no conflict of interest.

1

## Supporting information

As a service to our authors and readers, this journal provides supporting information supplied by the authors. Such materials are peer reviewed and may be re‐organized for online delivery, but are not copy‐edited or typeset. Technical support issues arising from supporting information (other than missing files) should be addressed to the authors.

Supporting Information

## Data Availability

The data that support the findings of this study are available in the supplementary material of this article.
