# Supplementary material for: PHRONESIS: A One‐Shot Approach for Sequential Assignment of Protein Resonances by Ultrafast MAS Solid‐State NMR Spectroscopy
Source: Chemphyschem. 2022 May 19;23(13):e202200127. doi: 10.1002/cphc.202200127 (PMC9400877; doi:10.1002/cphc.202200127)
Supplement: Supplementary file 1 — Supporting Information [file CPHC-23-0-s001.pdf]

ChemPhysChem

Supporting Information

PHRONESIS: A One-Shot Approach for Sequential Assignment of Protein Resonances by Ultrafast MAS Solid-State NMR Spectroscopy

Tata Gopinath,* Veliparambil S. Manu, Daniel K. Weber, and Gianluigi Veglia*

Supporting Information

Section 1: Selective GENETICS-AI pulses:

The new selective pulses are designed using a novel GENETICS-AI (Generator of Triply Compensated Pulses-Artificial Intelligence) algorithm, which uses an optimization network to evolve the RF pulse under a given NMR Hamiltonian with preferred operational specifications.[14] For the ^{13}C selective pulses, the NMR Hamiltonian is defined by ^{13}C chemical shifts, RF inhomogeneity, and CH couplings. The algorithm uses the known initial ^{13}C state (CO_z, CO_x, CX_z, or CX_x) and evolves the RF phase and pulse duration to achieve the desired final states for CX and CO spectral regions. In this case, CX (0 to 75 ppm) represents the CA and side-chain aliphatic ^{13}C peaks, and CO (160 to 185 ppm) represents the carbonyl spectral region. CX_z and CO_z represent the longitudinal ^{13}C polarization, whereas CX_x and CO_x correspond to transverse polarization. For a given operational fidelity, the algorithm optimizes the lowest RF pulse amplitude, which is kept constant throughout the pulse duration. In the current work, all the pulses were designed with ^{13}C RF amplitude of 16 kHz, and the RF carrier was set to 100 ppm during the simulations and experiments. Note that these selective pulses do not require frequency switching. In other words, for both CA and CO selective pulses, the ^{13}C offset is set to 100 ppm, and spectral selectivity is obtained via phase modulation of RF pulse. All the GENETICS-AI optimization protocols are coded in MATLAB and require approximately 5 hours on a personal computer equipped with an intel core i7 processor. The average operational fidelity of the pulses was set to 0.99. Similar to a 90° hard pulse, the new pulse affects the entire ^{13}C spectrum during the pulse evolution. However, the algorithm calculates the pulse phase modulation in such a way to affect only desired ^{13}C spectral region; whereas the polarization of the remaining spectral region goes back to the original state at the end of the pulse (Figure 1, main text).

The spectral profiles of selective pulses were simulated using the inbuilt NMR-SIM program of Bruker TOPSPIN (Figure S2). For selective 90° excitation of CX, the objective function of GENETICS-AI is to achieve the operations $C_z \rightarrow C_x$ for CX bandwidth and $C_z \rightarrow C_z$ for CO (Figure S2a). The spectral profile for CO excitation is shown in Figure S2b. For selective 90° flip-back pulse (Figures S2 c,d), the operations are $C_x \rightarrow C_z$ for the selected region and $C_z \rightarrow C_z$ elsewhere. These specifications are in accordance with the 3D PHRONESIS pulse sequence (Figure 2a). The excitation and flip-back pulses are applied on CA while keeping CO in the z-direction and vice-versa. The effect of selective pulses on side-chain ^{13}C resonances (0 to 40 ppm and 100 to 135 ppm) is not considered because these signals do not propagate in the 3D backbone correlation experiment, PHRONESIS.

Section 2: Broadband SIM-CP transfer:

Under fast MAS conditions, Hartmann-Hahn cross-polarization is achieved via ZQ (ν_1^H - $\nu_1^{C/N}$= ν_r or 2*ν_r) or DQ (ν_1^H + $\nu_1^{C/N}$ = ν_r or 2*ν_r) recoupling conditions. Here, the RF field amplitudes on ^1H,^{13}C, and ^{15}N are represented by ν_1^H, ν_1^C, and ν_1^N, whereas ν_r corresponds to the MAS frequency. To maximize the heteronuclear transfer efficiency, it is important to choose the RF amplitudes that do not satisfy the homonuclear rotational resonance conditions (i.e. ν_1=ν_r/2, ν_r or 2*ν_r) that would otherwise cause a signal loss.[15] In general, depending on the MAS frequency and RF probe specifications, either DQ or ZQ heteronuclear matching conditions are applied. In the current work with 65 kHz MAS, maximum signal intensities were obtained from ZQ

matching condition. To monitor the efficiency of broadband SIM-CP with respect to CP, we used ^{1}H detected 1D experiments shown in Figure S3. The CP edited ^{1}H spectra (shown in black) are obtained by replacing the SIM-CP block with HCX, or HCO, or HN CP. The optimized CP RF amplitudes are ν_1^C= 22 kHz, ν_1^N=21.3 kHz, and ν_1^H = 86 kHz. ^{13}C offset was set to 54 and 168 ppm for HCX and HCO CP, respectively. For broadband SIM-CP transfer, ^{13}C offset was set to 125 ppm, and high RF amplitude spin-lock pulses (ν_1^C=50 kHz ν_1^N=49.5kHz, and ν_1^H = 115kHz) were applied. For both CP and SIM-CP, ^{1}H ramp conditions were arrayed between 70-100% and 95-100%. Under ZQ matching conditions, we obtained the maximum signal using a 90-100% ^{1}H ramp. For CP-edited experiments, we also tested ZQ matching conditions using ν_1^C and ν_1^N values of 15-20 kHz, resulting in lower signal intensities than the CP-edited spectra of Figure S3. As shown in Figure S3, the intensities of CA-edited H^N spectra (shown in red) obtained from SIM-CP are similar to CP-edited spectra (black). Whereas the average intensities of CO and ^{15}N edited H^N spectra (shown in blue and green) obtained from SIM-CP are respectively 74 % and 70% with respect to CP edited spectra (shown in black). The corresponding ^{13}C and ^{15}N detected spectra obtained from CP and SIM-CP are shown in Figure S4. Note that we used 1.2 ms contact times for CA, CO, and ^{15}N CP and SIM-CP. Although (H)CANH and (H)NH show maximum intensities at around 300 μs (Figure S3a,c), in our experience, for uniformly labeled proteins at an effective temperature of 25ºC, the CP/SIM-CP contact times of 1 to 1.5 ms give optimal signal intensities for all the residues associated with structured and dynamic regions.

The loss of CO and ^{15}N SIM-CP signal intensities can be attributed to partial ^{1}H polarization sharing between ^{13}C and ^{15}N.[16] Also, note that the effective ^{1}H density surrounding CO and ^{15}N atoms is lower than CA carbons. As shown earlier, the CP and SIM-CP intensities are very similar for CA and side-chain carbon spectra for both slow and fast MAS experiments.[6, 17] We also compared the performance of broadband SIM-CP using DQ (double quantum) matching conditions, as well as with ^{13}C and ^{15}N RF ramp (Figures S12 and S13). Under these conditions, we found lower ^{13}C intensities than the ZQ matching condition with ^{1}H RF ramp (Figure S3). Interestingly, when the CO intensity from SIM-CP is lower, the corresponding ^{15}N signal intensity is higher (red spectra in Figures S12 and S13), indicating partial polarization sharing between CO and ^{15}N. Note that the relative efficiency of CP and SIM-CP can vary depending on the MAS rate, spectral dispersion (B_o field), and RF matching conditions. More recently, ^{1}H-edited SIM-CP experiments were also used to quantify the transferred and residual ^{1}H polarization during SIM-CP.[18] Note that unlike for slow or moderate MAS experiments (8 to 20 kHz), the truncation of ^{1}H-^{1}H couplings under fast MAS conditions narrows the CP/SIM-CP matching conditions.[19] The efficiency of broadband transfer might be improved using other polarization transfer schemes.[20]

Section 3: Broadband SPECIFIC-CP transfer (CA-N, CO-N, N-CA, and N-CO):

Under fast MAS conditions, heteronuclear polarization transfer between ^{13}C and ^{15}N is obtained by SPECIFIC-CP with CA (or CO) and ^{15}N spin-lock pulses that satisfy the DQ (double quantum) matching condition, $\nu_1^c+\nu_1^N=\nu_r$. Typically, a low RF amplitude (15 to 30 kHz) spin-lock pulse is applied at the center of CA (or CO) spectral region to selectively transfer the polarization between CA (or CO) and ^{15}N. A variety of NC recouping conditions can be obtained by moving the ^{13}C offset between CA and CO spectral regions. However, our goal is to maximize the CN and NC transfer efficiencies for both CA and CO; therefore, ^{13}C offset was centered between CA and CO (i.e., 115 ppm), and a relatively higher RF amplitude spin lock pulse (42 kHz) was applied

to cover both CA and CO spectral regions. Optimal signal intensities were obtained by arraying the ^{15}N RF according to DQ matching condition. Sensitivity comparison of broadband and selective SPECIFIC-CP is demonstrated in Figures S5 and S6, where the 1st and 2nd acquisitions, respectively, record the transferred and residual polarization. Using broadband SPECIFIC-CP, the intensities of transferred polarization pathways, CANtr, CONtr, NCAtr, and NCOtr, are respectively 103%, 73%, 55%, and 100% in comparison to selective SPECIFIC-CP. In contrast, the CA, CO, and ^{15}N residual polarization is 40%, 37%, and 10-12% (Figures S5d and S6d). The transfer efficiencies are also summarized in Figure S8b. We have previously shown that the residual ^{15}N polarization after NCA or NCO selective SPECIFIC-CP is 30 to 40% under slow and fast MAS conditions, similar to Figure S6b,c.[6, 10b] On the other hand, using broadband SPECIFIC-CP, ^{15}N polarization is simultaneously transferred to CA and CO, resulting in lower ^{15}N residual polarization (Figure S6d). Note that at higher spinning speeds (~100 kHz), it might be possible to increase broadband SPECIFIC-CP efficiency using high RF ^{13}C spin-lock pulses, according to DQ matching conditions.

Section 4:Bidirectional DREAM transfer (CA-CO and CO-CA):

Homonuclear CC DREAM transfer is obtained by RF amplitude sweep (ramp or tangential ramp) through HORROR recoupling condition ($\nu_{1C}=\nu_r/2$).[2b, 15] At slow and moderate spinning speeds (10 to 30 kHz), DREAM requires a low RF amplitude spin-lock pulse, and the resulting transfer efficiency is very sensitive to ^{13}C offset and RF amplitude.[21] At fast MAS rates, the broader RF matching conditions of the DREAM transfer are facilitated by high RF amplitude spin-lock pulses (ν1C=νr/2), enabling simultaneous (or bidirectional) optimization of the CACO and COCA transfers. The bidirectional DREAM transfer is optimized by using 1D HCACONH and HCOCANH pulse sequences shown in Figure S7a. DREAM was tested with several ramp conditions (70-100% to 95-100%), and 80-100% gave the maximum transferred signal. We incorporated the selective CA and CO pulses before and after the DREAM to monitor the CACO and COCA pathways. When DREAM was applied at the CA region (54 ppm), it is possible to choose an RF amplitude (represented by the dotted line in Figure S7b) that maximizes CACO transfer while achieving almost 80% COCA transfer in comparison to on-resonance COCA DREAM (Figure S7c). Similarly, bi-directional DREAM can also be obtained by maximizing COCA transfer while achieving 70% CACO (dotted line in Figure S7c). In the current work, we used a 600 MHz spectrometer. The CA and CO regions are separated by an average of 16 kHz bandwidth, which is smaller than the DREAM RF amplitude (~30 kHz), enabling efficient bidirectional CACO and COCA transfer. However, for larger CACO bandwidths (high field spectrometers), optimizing the offset of DREAM spin-lock pulse may be necessary to achieve bidirectional DREAM transfer.[21] A broadband DREAM transfer was demonstrated using high RF amplitude spin lock periods by combining XIX and DREAM RF profiles under moderate MAS conditions.[22] Note that under very fast spinning speeds (~100 kHz) with improved T2 relaxation times, DREAM can be replaced with bidirectional CC INEPT (*i.e.*, CACO and COCA) transfer.[23]

Section 5: Evaluation of water peak during 3D PHRONESIS:

In the PHRONESIS experiment (Figure 2a), the 1° pulse applied on ^{1}H channel retains 99.99% z-polarization (cos1°=0.99), and only 0.01% transverse polarization (sin1° = 0.02) is detected. Hence, there is almost no impact on the z-polarization. For highly concentrated water molecules, 1° pulse is sufficient to produce enough transverse signal for the detection (Figure 2b, and S11a). The 1D proton spectra of crystalline GB1 (Figure 2b) consists of two peaks corresponding to water (4.7

ppm) and methyl protons (1.4 ppm) of MPD (methylpentanediol) that is used for crystallization.[24] All the 1D spectra acquired during the 3D showed only incremental peak shifts caused by magnet (Bo field) drift. We did not see any abnormal peak shifts which would arise from the spectrometer instabilities or RF heating (Figure S11). We found almost equal intensities and line widths for all the 1D peaks obtained from 3D PHRONESIS. In contrast to slow MAS, fast MAS pulse sequences only require low RF amplitude (5-10kHz) decoupling periods and therefore do not cause RF heating even under multiple acquisition periods. The gradual peak shift of water spectra recorded during the 3D PHRONESIS experiment (52 h) matches with spectrometer $\mathbf{B}_o$ drift. Due to broad water line widths (0.17 ppm) caused by free and bound water molecules, it was not possible to unambiguously monitor the Bo drift during the 3D experiment. Nonetheless, water (or solvent) spectra offer insights into understanding the spectrometer and sample conditions during the 3D experiment.

Table S1: 3D PHRONESIS experimental parameters for Figure 1 (main text)

Spectrometer: 600 MHz Bruker NEO; Probe: 1.3 mm fast MAS; Sample: ~2 mg crystalline GB1

MAS frequency: 65 kHz; RF coil Temperature: -18ºC; Effective sample temperature: 25 ºC;

RF parameters

RF pulse/spin- lock	^{1}H (offset: 4.8 ppm)		^{13}C (offset: 100 ppm)			^{15}N (offset: 120 ppm)	
	RF amplitude	Duration	Offset (ppm)	RF amplitude	Duration	RF amplitude	Duratio n
90º pulse	200 kHz	1.25 µs		83.3 kHz	3.0 µs	83.3 kHz	3.0 µs
180º	200 kHz	2.5 µs		83.3 kHz	6.0 µs	83.3 kHz	6.0 µs
1º	5 kHz	0.5 µs					
90º CA excitation				16.14 kHz	109 µs		
90º CA flip back				16.14 kHz	102 µs		
90º CO excitation				16.14 kHz	59 µs		
90º CO flip back				16.14 kHz	88 µs		
180º CA (Q3)			54	5.02 kHz	700 µs		
180º CO (Q3)			168	5.02 kHz	700 µs		
SIM-CP (ZQ recoupling)	114 kHz 90% - 100%	1.2 ms	125	50 kHz	1.2 ms	49.5 kHz	1.2 ms
SPECIFIC-CP (DQ recoupling)			115	41.7 kHz	9 ms	22.2 kHz 85% - 100%	9 ms
DREAM (DQ recoupling)			54	30.5 kHz 80% -100%	9 ms		
WALTZ16 decoupling	10 kHz	t1 and t2				10 kHz	t3
NH CP	84.2 kHz 90 to 100%	400 µs				22.7 kHz	400 µs
Water suppression	30 kHz	100 ms					
RFDR recoupling		738 µs					

Acquisition parameters

Parameter	t_1 (States-TPPI)			t_2 (States-TPPI)	t_3 (DQD)
	CA (54 ppm)	CO (168 ppm)	N (120 ppm)	N (120 ppm)	H (4.8 ppm)
dw (dwell time)	225 µs	450 µs	450 µs	450 µs	5 µs
ni (no. of increments)	36	18	18	18	1664
n (no. of loops)	1	2	2	1	
T (total evolution time) =dw*(ni-1)	7.88 ms	7.65 ms	7.65 ms	7.65 ms	8.32 ms Dig. mode: “baseopt”

Recycle delay (d1): 2 s; No. of t3 acquisition periods (nbl): 4; No. of scans (ns): 2;
No. of Hadamard phase encoded data sets:16; Total experimental time: 52 h.

Table S2: GB1 assignments obtained from the 3D PHRONESIS experiment.

Residue	H(i)	N(i)	Cα(i) (H)CANH	Cα(i-1) (H)CA(CO)NH	C’(i-1) (H)CONH	C’(i) (H)CO(CA)NH	N(i-1) (H)N(CACO)NH	N(i+1) (H)N(COCA)NH	Hα (H)CAN(H^N)H/ (H)CON(H^N)H
M1	-	-	-	-	-	-	-	-	-/-
Q2	8.534	125.957	55.845	-	171.144	174.87	-	-	3.904/-
Y3	9.29	122.843	-	55.975	-	174.913	-	-	-/-
K4	9.375	123.235	-	-	-	173.252	123.364	126.984	-/-
L5	9.313	127.077	53.005	-	173.232	-	123.412	-	5.899/-
I6	9.157	126.621	59.977	53.161	-	-	-	-	4.756/-
L7	9.404	127.418	54.761	59.934	-	-	-	-	-/-
N8	9.156	125.522	-	-	174.839	176.352	-	-	-/-
G9	8.333	109.717	44.619	50.702	176.197	173.177	-	121.34	-/-
K10	9.977	121.328	59.216	44.378	173.086	178.95	109.889	106.975	-/-
T11	9.095	107.016	62.059	59.21	178.894	173.156	121.599	127.823	4.054/4.204
L12	7.445	127.774	54.513	62.002	173.116	173.862	106.43	123.633	-/-
K13	9.038	123.596	53.462	-	173.744	-	-	105.963	5.556/5.814
G14	8.755	106.057	45.018	53.416	175.676	170.952	123.526	121.647	-/3.195, 3.725
E15	8.975	121.689	53.888	44.978	171.021	173.903	106.046	-	-/5.343
T16	9.012	115.854	60.281	53.891	-	171.742	121.788	116.225	-/-
T17	8.443	116.339	60.324	60.291	171.825	174.056	116.049	116.435	-/-
T18	9.298	116.461	61.531	60.277	-	-	116.167	-	5.233/-
E19	7.972	125.644	54.474	61.498	171.221	175.574	116.468	125.39	-/-
A20	9.419	125.129	-	-	175.575	177.497	-	116.297	-/-
V21	8.757	116.435	63.789	50.79	-	174.902	-	116.21	-/-
D22	7.431	115.656	52.508	63.76	174.769	175.074	116.014	123.423	-/-
A23	9.29	123.176	-	52.589	-	-	115.84	121.173	-/-
A24	8.163	120.82	54.594	54.624	-	181.259	123.605	117.52	-/-
T25	8.579	117.46	67.601	54.817	181.192	-	-	-	3.945/-
A26	7.427	124.169	55.109	67.689	175.662	177.268	117.358	116.959	-/-
E27	8.757	116.636	59.281	-	-	177.457	-	-	-/-
K28	7.143	117.563	60.267	59.419	177.608	178.887	117.309	119.01	-/-
V29	7.683	118.996	66.514	60.208	178.668	178.389	117.87	118.931	3.799/-
F30	8.774	118.846	-	66.555	-	-	119.292	-	-/-
K31	9.165	121.007	60.116	-	179.272	-	-	-	4.753/-
Q32	8.022	121.382	58.921	60.182	-	177.351	-	121.096	-/-
Y33	8.774	121.268	61.659	58.823	177.413	-	121.524	-	-/4.592

A34	9.24	122.806	-	61.584	178.34	-	-	118.643	-/3.963
N35	8.622	118.721	-	56.067	179.442	-	-	-	-/4.347
D36	9.188	121.628	-	57.193	-	175.837	-	-	-/-
N37	7.409	115.223	53.583	55.981	175.831	173.953	121.942	108.387	4.624/4.669
G38	8.051	108.417	46.784	53.543	174.008	173.897	115.377	122.27	-/4.422, 3.751
V39	8.386	122.078	61.879	46.799	173.834	174.908	108.581	131.082	-/4.446
D40	9.14	131.134	52.792	-	174.901	174.932	122.204	108.472	-/4.388
G41	8.063	108.463	45.158	52.752	174.974	172.661	131.186	119.655	-/5.238, 4.782
E42	8.741	119.641	55.192	45.267	172.61	177.705	108.471	125.649	-/5.281
W43	9.346	125.633	57.594	-	177.652	-	119.639	-	5.292/5.402
T44	9.145	109.446	60.964	57.591	176.83	173.701	125.804	119.128	-/-
Y45	9.458	119.083	57.766	60.926	173.726	171.843	109.762	126.826	-/4.269
D46	7.659	126.885	50.953	57.916	171.706	-	-	-	4.98/-
D47	9.036	124.159	-	51.116	175.931	-	-	-	-/-
A48	8.642	119.252	53.962	54.396	177.049	-	-	104.312	4.421/4.489
T49	7.131	104.248	60.571	53.834	178.983	175.415	119.237	119.703	5.153/4.98
K50	8.204	119.587	55.281	-	175.457	-	104.231	112.617	-/-
T51	7.568	112.67	62.587	55.365	175.234	174.073	119.747	130.311	5.024/-
F52	10.826	130.317	56.602	62.57	174.128	175.625	112.766	112.514	-/-
T53	9.399	112.482	60.401	56.523	175.666	172.041	129.42	118.632	-/-
V54	8.298	118.513	58.628	60.49	171.909	172.267	112.704	124.422	4.151/-
T55	8.535	124.362	61.358	58.718	172.428	173.808	118.712	131.427	5.062/-
E56	8.044	131.407	57.58	-	173.86	180.322	124.627	-	4.764/-

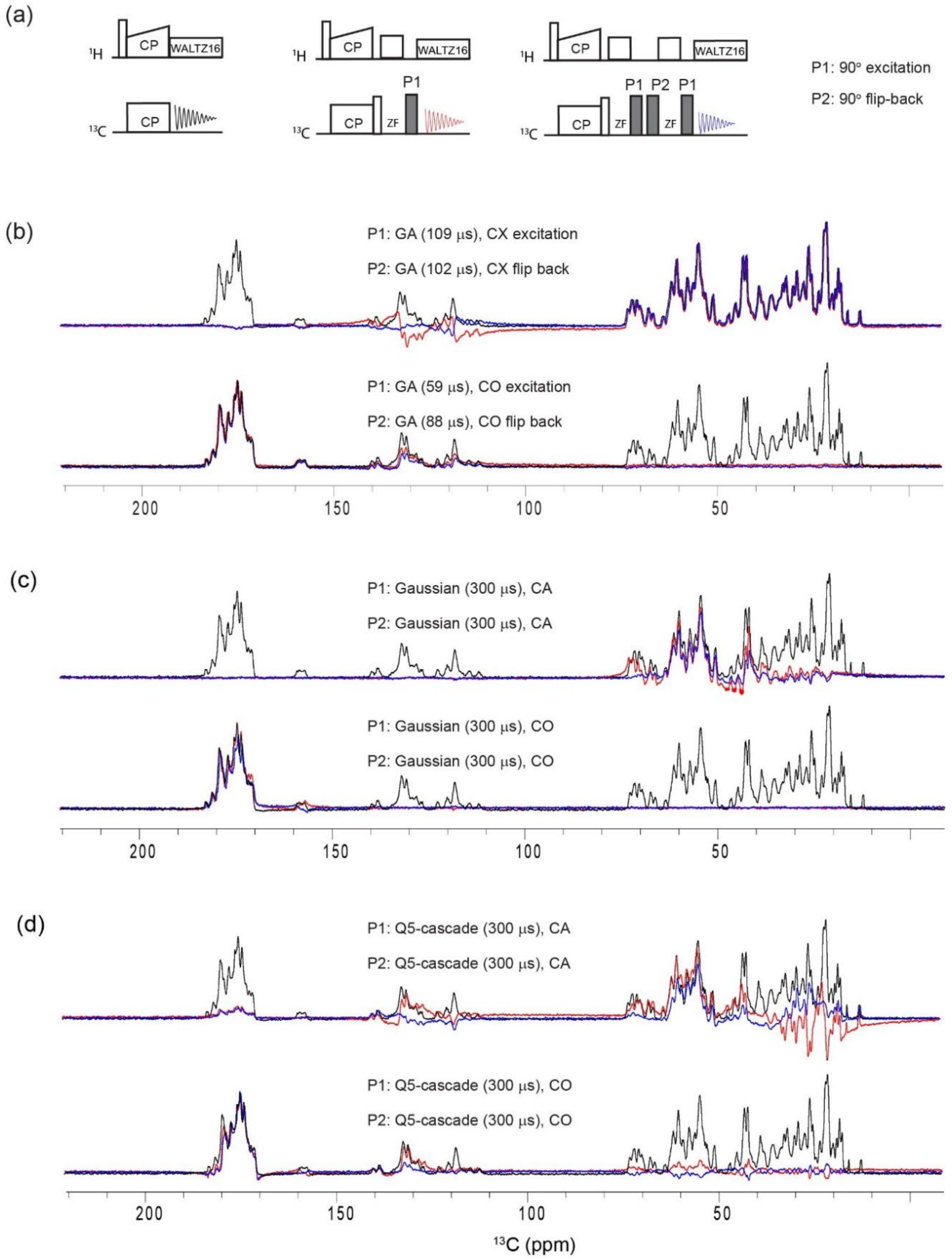

Figure S1: (a) ^{13}C detected experiments for testing the efficiency of selective GENETICS-AI, Gaussian, and Q5 cascade pulses. CX represents CA and aliphatic side-chain peaks (~ 0.to 75 ppm), whereas CO represents carbonyl spectral region (~160 to 185 ppm). All the spectra were acquired with CP parameters, ν_{1H} = 114 kHz, ν_{1C}=50 kHz, and ^{13}C offset was set to 125 ppm. (b-d) The reference CP spectra (black) contains all the peaks. The spectra shown in red were obtained from CP followed a 90° hard pulse to create ^{13}C z-polarization, selectively (CX or CA or CO) excited using P1 pulse. The spectra shown in blue were obtained by flipping the z-polarization using three consecutive 90° excitation (P1), flip-back (P2), and excitation (P1) selective pulses. The spectra obtained from GENETICS-AI pulses (b) show zero signal loss with respect to the reference CP spectrum. Whereas for selective spectra obtained from Gaussian and Q5 pulses, we found a signal loss of 10 to 40%. All the GENETICS-AI pulses were implemented with a ^{13}C offset set to 100 ppm and 16.11 kHz RF amplitude. Gaussian and Q5 selective pulses were implemented with ^{13}C offset set to 54 ppm for CA, 168 ppm for CO, and RF amplitudes (3-7 kHz) were optimized to maximize the signal intensities.

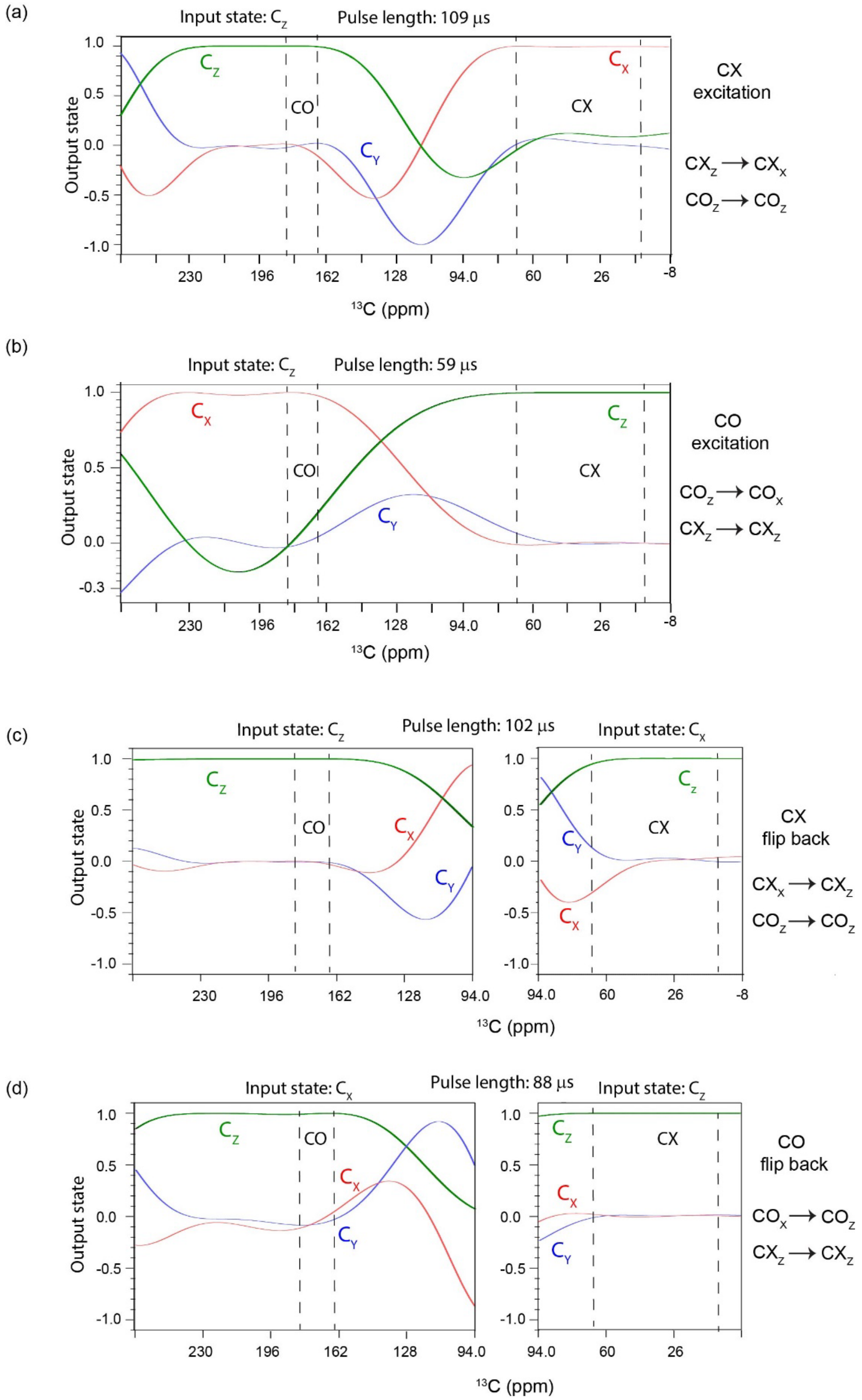

Figure S2: Simulated ^{13}C spectral profiles of 90° GENETICS-AI (GAI) pulses for a 600 MHz spectrometer. (a) CX excitation, (b) CO excitation, (c) CX flip back, and (d) CO flip back. The spectral profiles of GENETICS-AI pulses are obtained from the NMR-SIM program of BRUKER TOPSPIN, using longitudinal (C_Z) and transverse (C_X) initial states. In each of the excitation (C_Z to C_X) and flip-back (C_X to C_Z) profiles, only the input state of either CX (0 to 75 ppm) or CO (165 to 185 ppm) is affected. As shown in Figure 1 (main text), during the pulse, the entire 13C spectrum is excited; however, only the desired spectral region is affected at the end of the pulse. For example, at the end of CX excitation and flip-back pulses, the z-polarization of the CO region remains along z-direction and vice-versa. These pulses were tested using 1D ^{13}C detected experiments (Figure S1b) and incorporated in the 3D PHRONESIS experiment (Figure 2a).

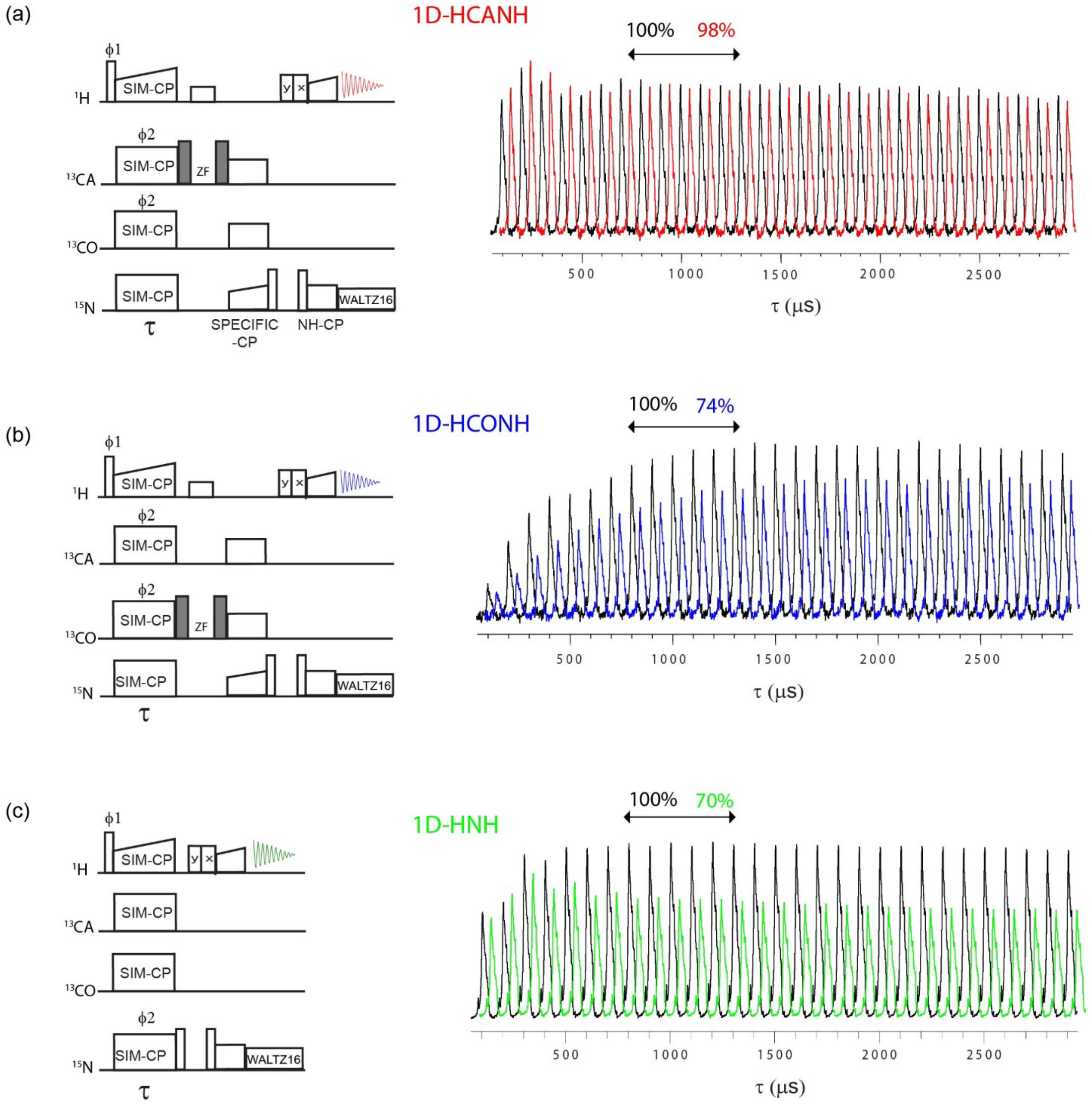

Figure S3: Comparison of CP and broadband SIM-CP using CA, CO, and ^{15}N edited 1D experiments HCANH, HCONH, and HNH, respectively. For SIM-CP, ^{13}C offset was set to 125 ppm, and optimized RF amplitudes are given by ν_{1H} = 114 kHz, ν_{1C}=50 kHz, and ν_{1N}=49.5 kHz. During SIM-CP, polarization is simultaneously transferred from ^{1}H spin bath to CA, CO, and ^{15}N (*i.e.*, HCA, HCO, and HN). The corresponding CP (*i.e.*, HCA or HCO or HN) edited ^{1}H spectra (shown in black) were acquired with optimized matching conditions, ν_{1H} = 86 kHz, ν_{1C}=22 kHz, andν_{1N}=21.3 kHz, and ^{13}C offset was set to 54 ppm for HCANH, and 168 ppm for HCONH. For both CP and SIM-CP, ^{1}H RF was ramped from 90 to 100%. The RF parameters for SPECIFIC-CP and NH-CP are reported in Table 1. The gray rectangles represent selective GENETICS-AI pulses.

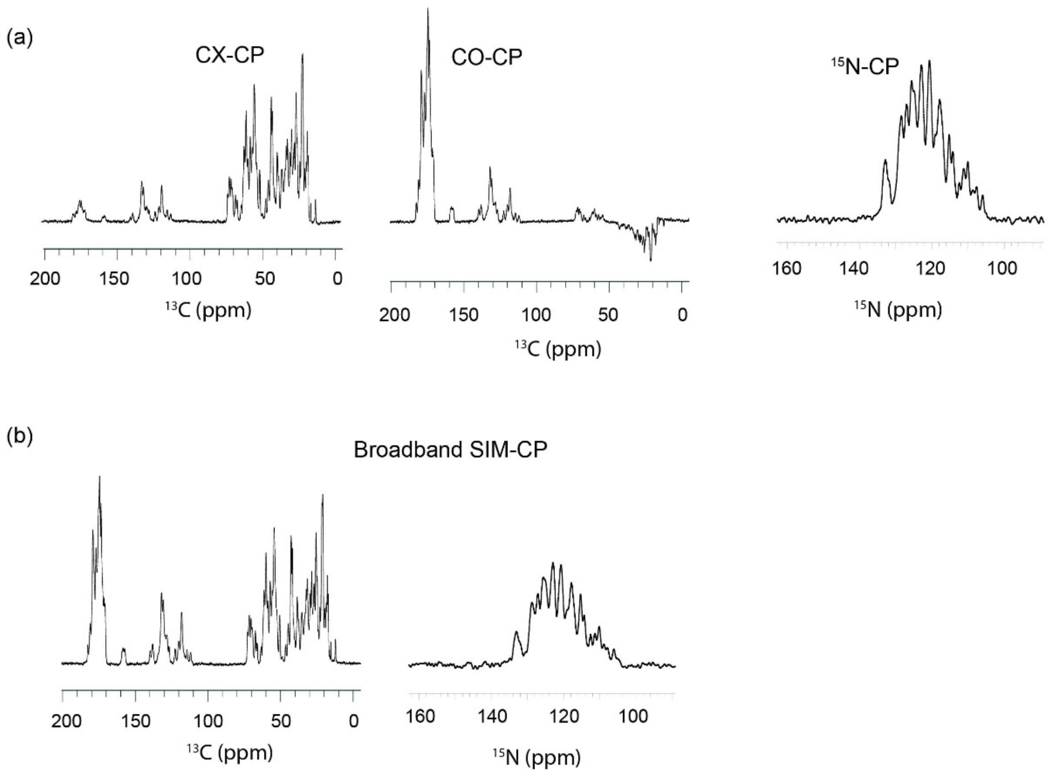

Figure S4: ^{13}C and ^{15}N detected 1D spectra obtained from broadband SIM-CP and CP, using 1.2 ms contact time (τ). The SIM-CP spectra were simultaneously recorded using dual receiver (^{13}C and ^{15}N) acquisition. CX, CO, and ^{15}N optimized CP spectra were acquired in three separate experiments. All the RF parameters for CP and SIM-CP are identical to Figure S3.

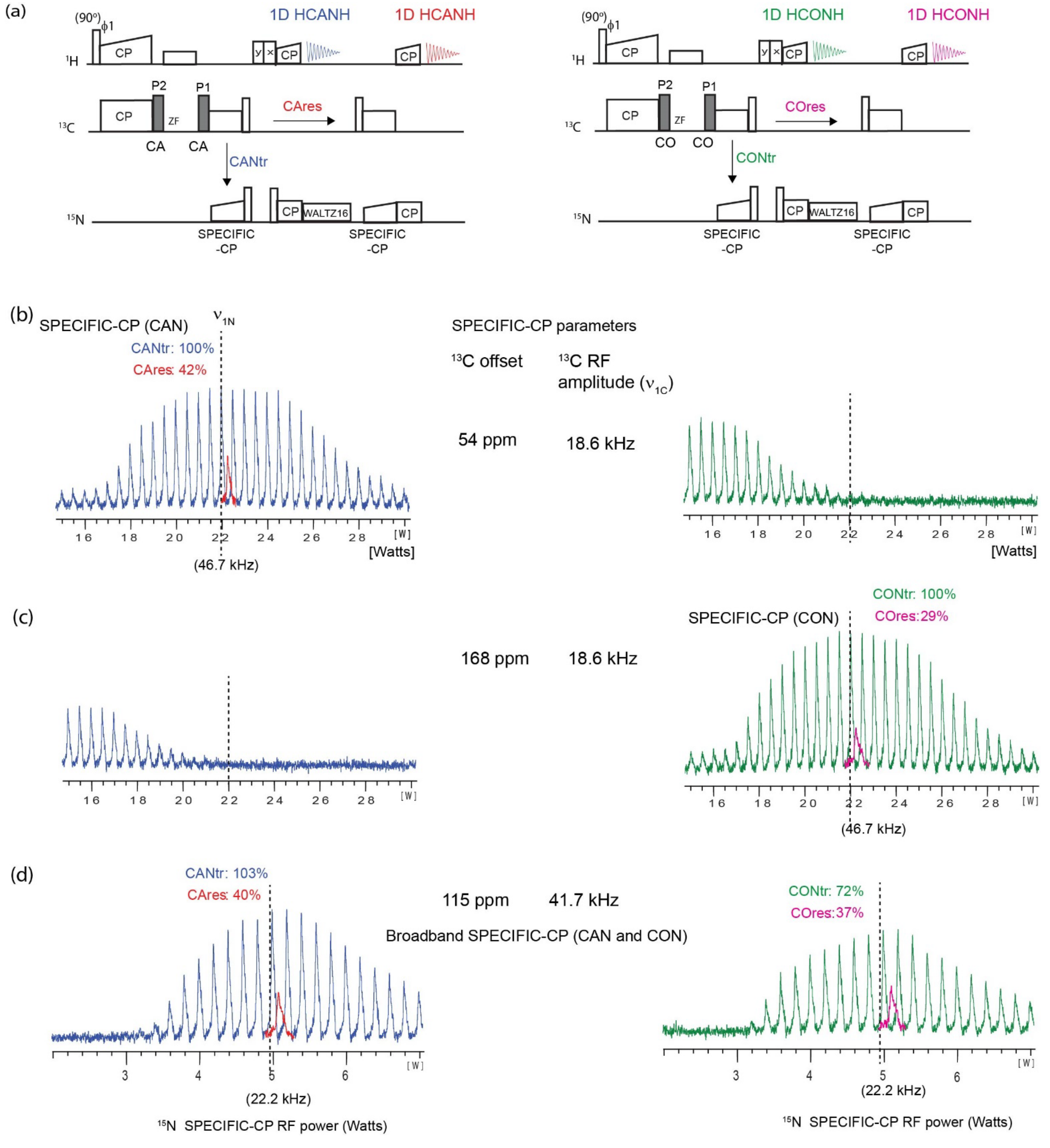

Figure S5: (a) one-dimensional HCANH and HCONH pulse sequences for optimizing the SPECIFIC-CP transfer between ^{13}C and ^{15}N. The CA or CO's polarization is selected via GENETICS-AI selective pulses (P1 and P2) and z-filter and then transferred to amide protons using CN SPECIFIC-CP and NH CP transfer periods. After 1st acquisition, the residual CA or CO polarization is detected in the 2nd acquisition period using identical CN and NH transfer periods. (b) Maximum HCANH transfer is obtained at ν_{1c}=18.6, and ν_{1N}=46.7 kHz, which approximately satisfies the DQ recoupling condition ($\nu_{1c}+\nu_{1n} \sim \nu_r$) with MAS frequency of ν_r=65 kHz. Whereas HCONH shows no signal (green spectrum dotted line). (c) Selective CON SPECIFIC-CP optimization using HCONH sequence and HCANH show zero signal (blue spectrum dotted line). (d) Broadband CAN and CON SPECIFIC-CP with ^{13}C offset of 115 ppm and optimized RF amplitudes, ν_{1c}= 41.7 kHz and ν_{1N} = 22.2 kHz. All the spectra were recorded with a 9 ms SPECIFIC-CP mixing period using a ^{15}N RF ramp 85% to 100%. For clarity, the residual polarization is only shown for optimal transfer conditions. The 1st and 2nd acquisitions were separately acquired using “popt“ (Bruker Topspin) by varying ^{15}N RF power.

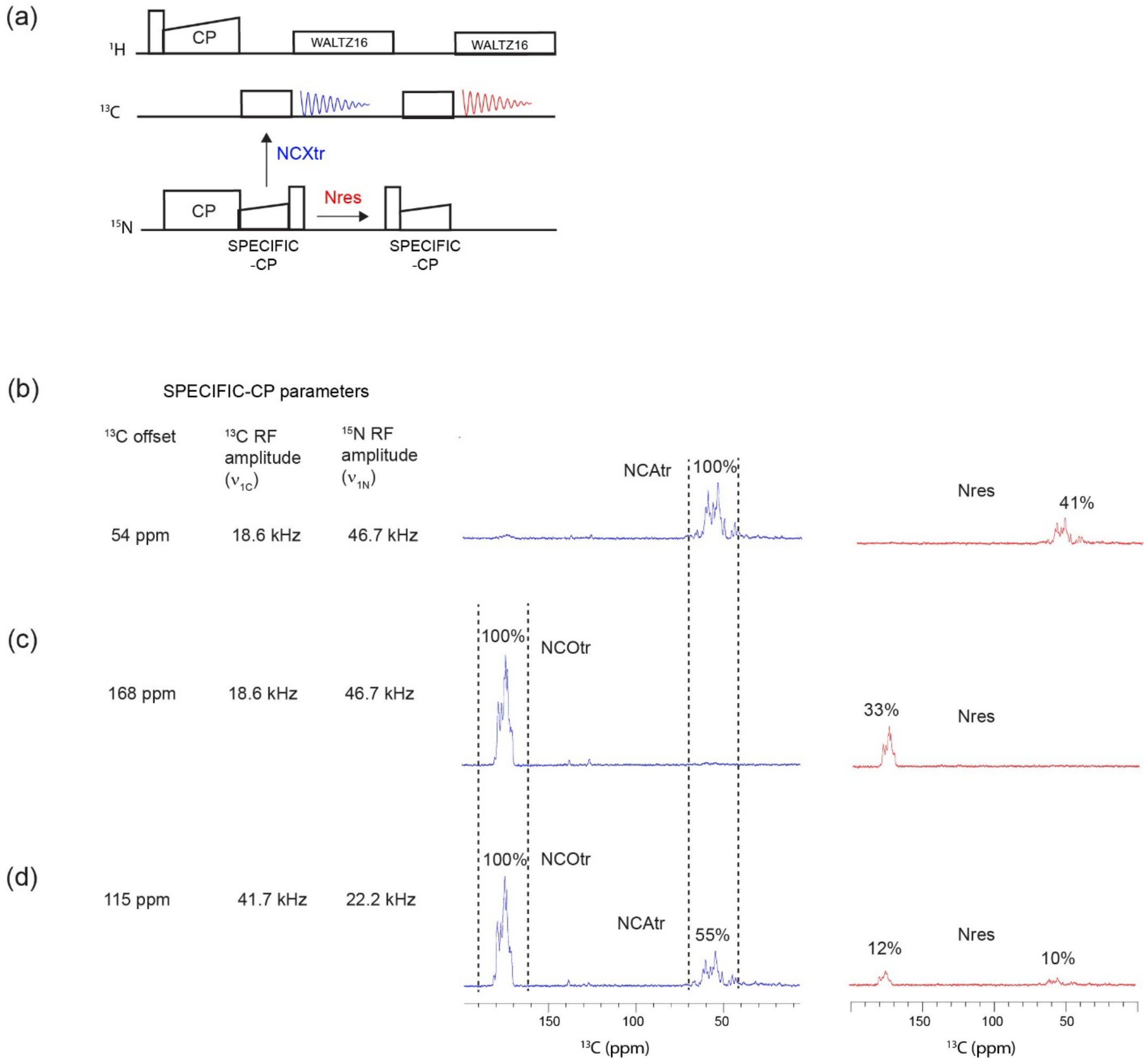

Figure S6: (a) Pulse sequence used for monitoring the NC transferred and ^{15}N residual polarization, respectively detected in 1st and 2nd acquisitions. The optimal SPECIFIC-CP RF parameters are obtained from Figure S5. (b), (c) Selective NCA and NCO transfer (blue spectra), and corresponding ^{15}N residual polarization (red spectra). (d) Spectra obtained from broadband polarization transfer and the corresponding residual polarization.

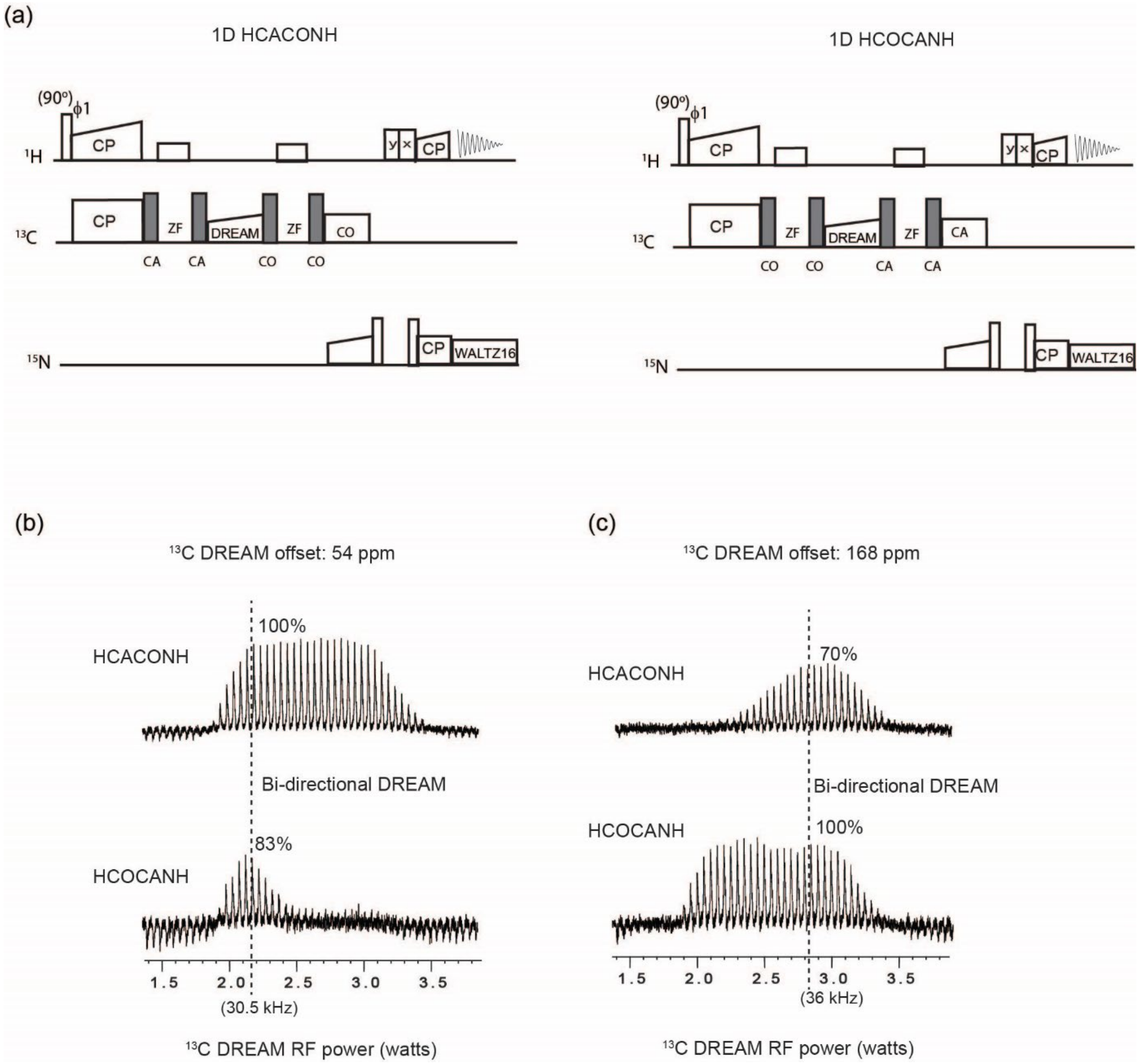

Figure S7: (a) One-dimensional HCACONH and HCOCANH pulse sequences used for optimizing CACO and COCA DREAM transfer. During CP, broadband polarization transfer from ^{1}H spin bath to CA and CO is obtained by setting the ^{13}C offset to 125 ppm and RF amplitudes, ν_{1H} = 114 kHz and ν_{1C}=50 kHz. Selective CA and CO GENETICS-AI pulses were used before and after the DREAM to select either CACO or COCA transferred polarization. After DREAM, CAN and CON SPECIFIC-CP were used for HCACONH and HCOCANH pulse sequences, respectively. HCACONH and HCOCANH spectra were acquired by varying the DREAM RF power. During the DREAM spin-lock pulse, the ^{13}C offset was set to 54 ppm (b) and 168 ppm (c). DREAM was implemented with a 9ms mixing period using an RF amplitude ramp 80% to 100%. The dotted lines correspond to bi-directional DREAM transfer optimized for maximum (b) CACO and (c) COCA transfer.

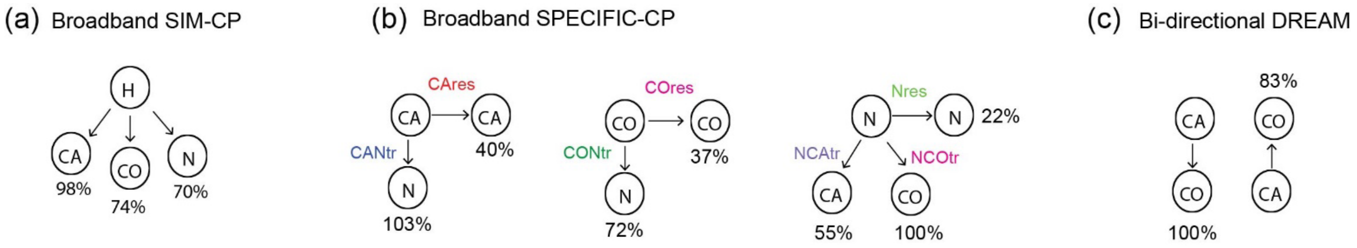

Figure S8: Summary of relative transfer efficiencies of broadband SIM-CP, SPECIFIC-CP, and bi-directional DREAM, as reported in Figures S3-S7. The intensities were normalized with respect to respective CP, SPECIFIC-CP, and DREAM signal intensities as 100%.

Hadamard decoding

Scan no.	(H)CONH<br>(H)CONhH	(H)CANH<br>(H)CAN(H)H<br>(H)CAcoNH	(H)NNH<br>(H)NN(H)H<br>(H)NcacoNH										(H)COcaNH		(H)NcocaNH	
1	1	1	1	1	1	1	1	1	1	1	1	1	1	1	1	1
2	1	-1	1	-1	1	-1	1	-1	1	-1	1	-1	1	-1	1	-1
3	1	1	-1	-1	1	1	-1	-1	1	1	-1	-1	1	1	-1	-1
4	1	-1	-1	1	1	-1	-1	1	1	-1	-1	1	1	-1	-1	1
5	1	1	1	1	-1	-1	-1	-1	1	1	1	1	-1	-1	-1	-1
6	1	-1	1	-1	-1	1	-1	1	1	-1	1	-1	-1	1	-1	1
7	1	1	-1	-1	-1	-1	1	1	1	1	-1	-1	-1	-1	1	1
8	1	-1	-1	1	-1	1	1	-1	1	-1	-1	1	-1	1	1	-1
9	1	1	1	1	1	1	1	1	-1	-1	-1	-1	-1	-1	-1	-1
10	1	-1	1	-1	1	-1	1	-1	-1	1	-1	1	-1	1	-1	1
11	1	1	-1	-1	1	1	-1	-1	-1	-1	1	1	-1	-1	1	1
12	1	-1	-1	1	1	-1	-1	1	-1	1	1	-1	-1	1	1	-1
13	1	1	1	1	-1	-1	-1	-1	-1	-1	-1	-1	1	1	1	1
14	1	-1	1	-1	-1	1	-1	1	-1	1	-1	1	1	-1	1	-1
15	1	1	-1	-1	-1	-1	1	1	-1	-1	1	1	1	1	-1	-1
16	1	-1	-1	1	-1	1	1	-1	-1	1	1	-1	1	-1	-1	1

Figure S9: Decoding of 3D PHRONESIS pathways using respective columns of sixteen dimensional Hadamard matrix. The sixteen interleaved scans are obtained by switching the phases of ϕ_1^H, ϕ_2^H, ϕ_3^H, and ϕ_4^H (Figure 2a). The decoding script is incorporated in NMRpipe processing files (Figure S19).

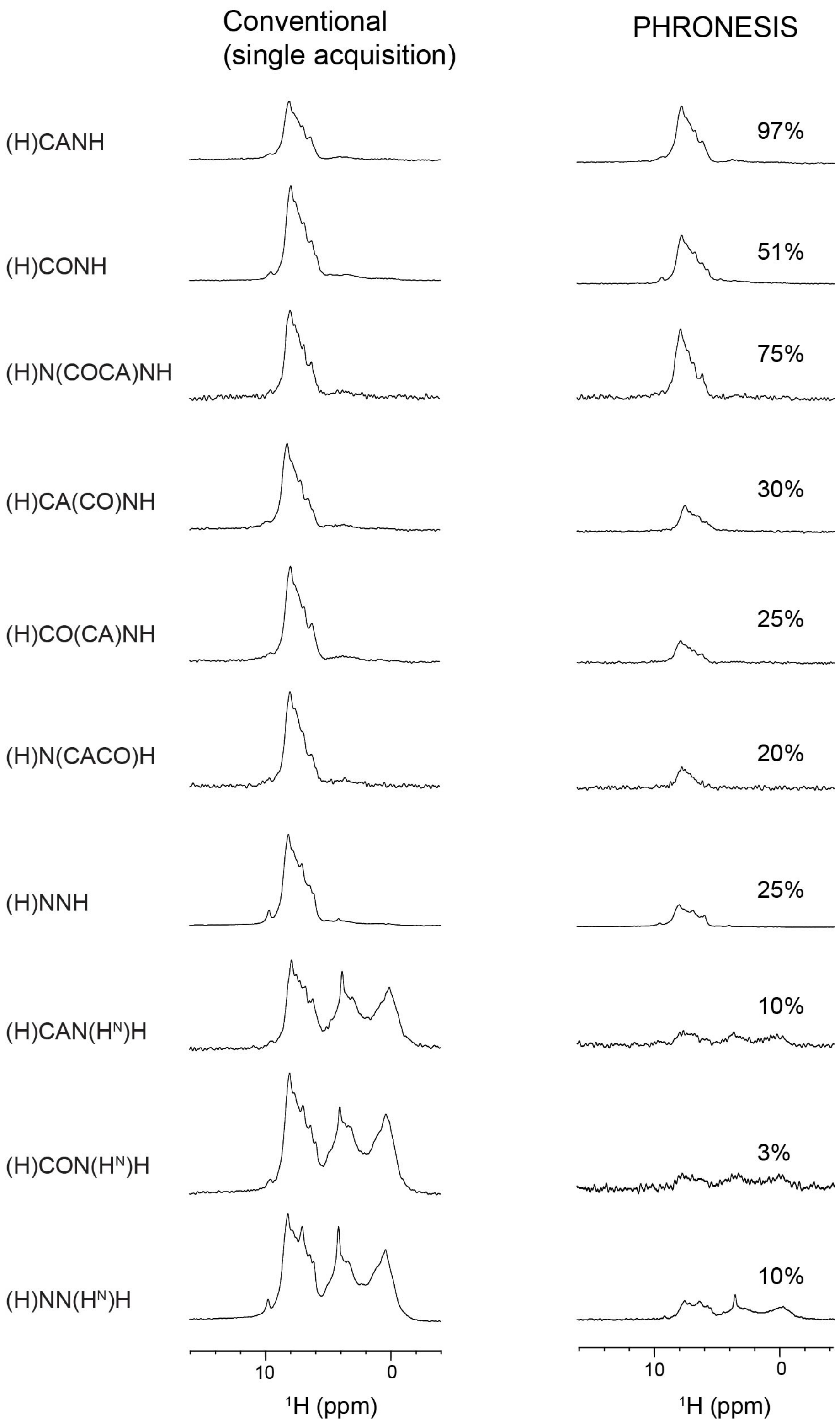

Figure S10: Comparison of 1D ^{1}H spectral intensities obtained from the 1st increment (t1=t2=0) of single acquisition and PHRONESIS 3D experiments using 32 scans. Single acquisition spectra were acquired separately using conventional 3D pulse sequences. The PHRONESIS spectra were simultaneously acquired from the pulse sequence of Figure 2a (main text), followed by Hadamard decoding of the data sets. Each PHRONESIS spectrum is normalized relative to the corresponding single acquisition spectral intensity as 100%.

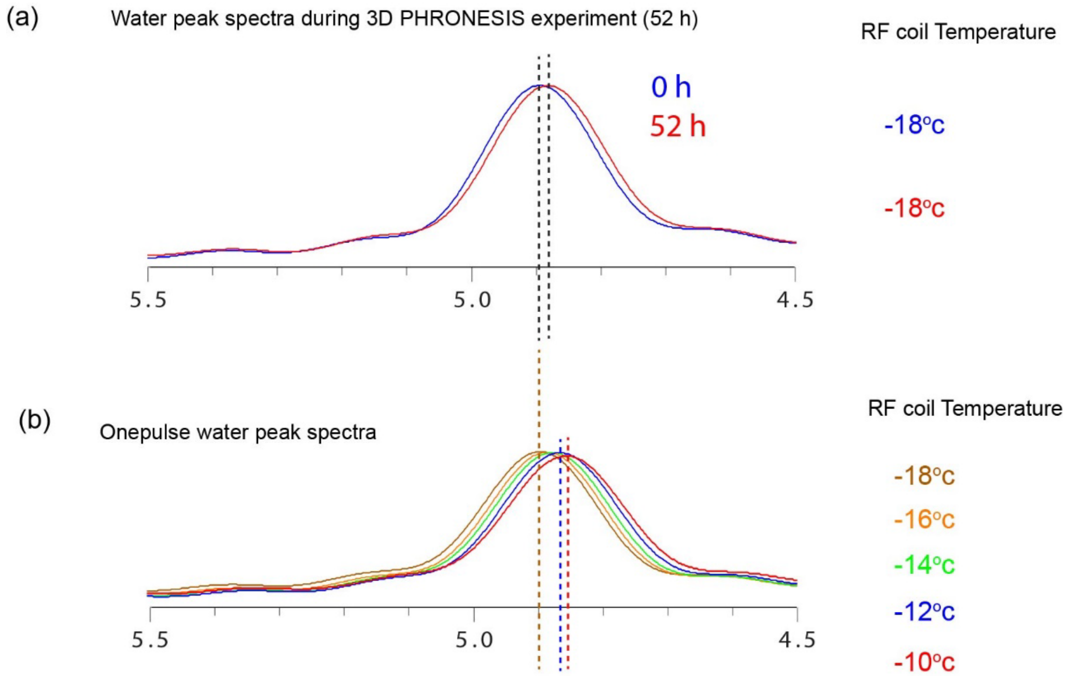

Figure S11: (a) Initial and final ^{1}H spectra of water peak recorded from the 1st acquisition of 3D PHRONESIS experiment (Figure 2b). The difference in chemical shift position matches with expected Bo drift during 52 h of the 3D PHRONESIS experiment. (b) 1D spectra were acquired using one pulse ^{1}H experiment at different temperatures, demonstrating the water peak shift with temperature variation.

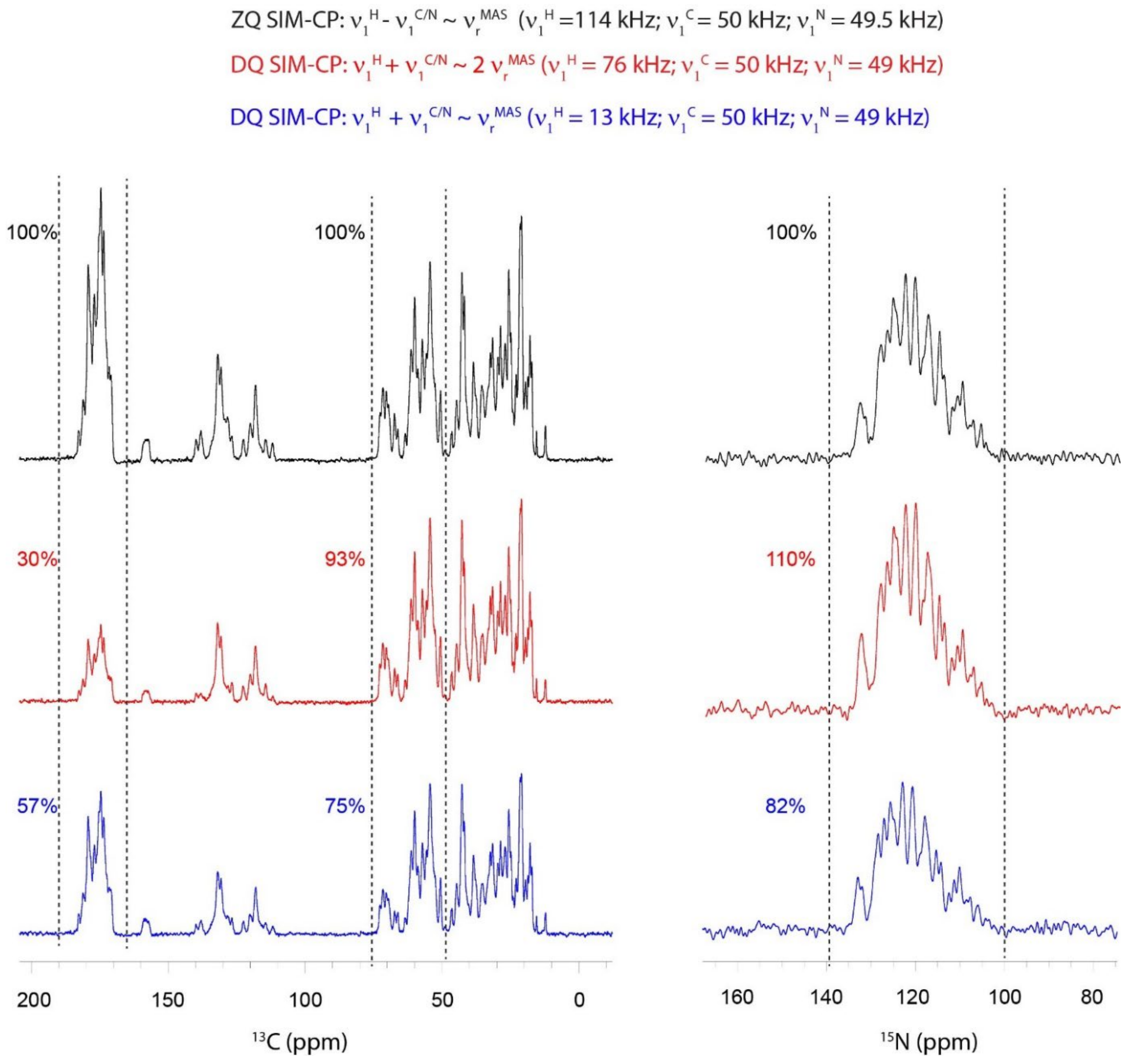

Figure S12: ^{13}C and ^{15}N SIM-CP spectra acquired using ZQ (zero quantum) and DQ (double quantum) Hartmann-Hahn matching conditions. The spectra were recorded with dual receiver acquisition under ^{1}H decoupling, using SIM-CP contact time of 1.2 ms. The matching conditions were optimized by arraying the ^{1}H and ^{15}N RF amplitudes, whereas ^{13}C RF was fixed to 50 kHz. The spectra demonstrate the high efficiency of broadband SIM-CP using the ZQ matching condition.

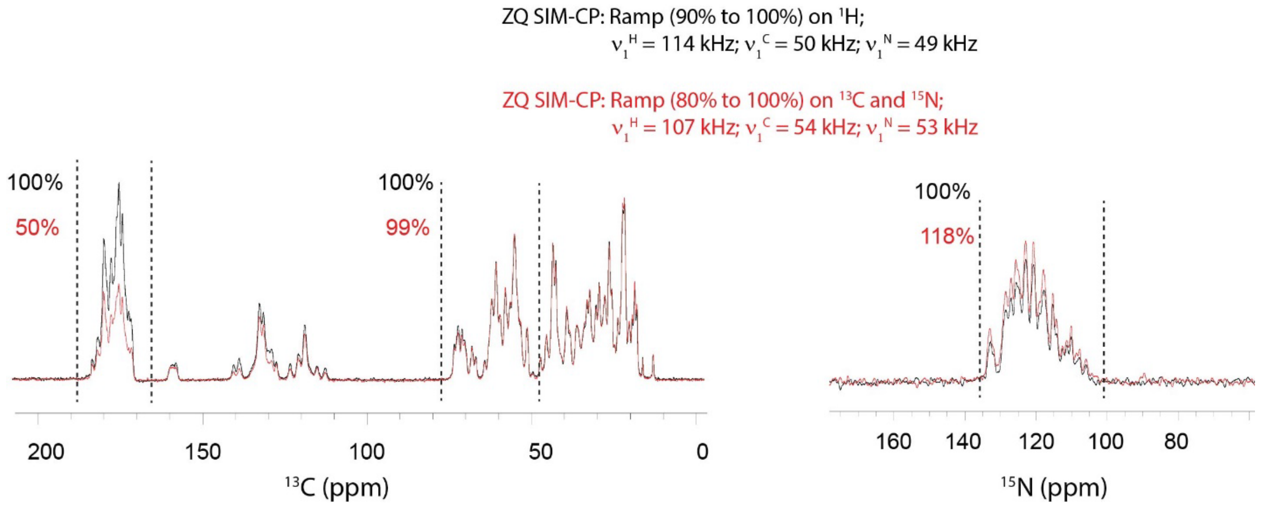

Figure S13: Comparison of ^{13}C and ^{15}N SIM-CP spectra obtained from ZQ matching condition with ^{1}H RF ramp (black) and ^{13}C and ^{15}N ramps (red). The spectra were recorded with dual receiver acquisition under ^{1}H decoupling, using SIM-CP contact time of 1.2 ms. The matching conditions were optimized by arraying the ^{1}H and ^{15}N RF amplitudes. Several ramp conditions were also tested to maximize the intensities. Optimal signal intensity was achieved with 80% to 100% RF ramp on ^{13}C and ^{15}N. Whereas for ^{1}H, 90% to 100% ramp gave maximum signal intensity.

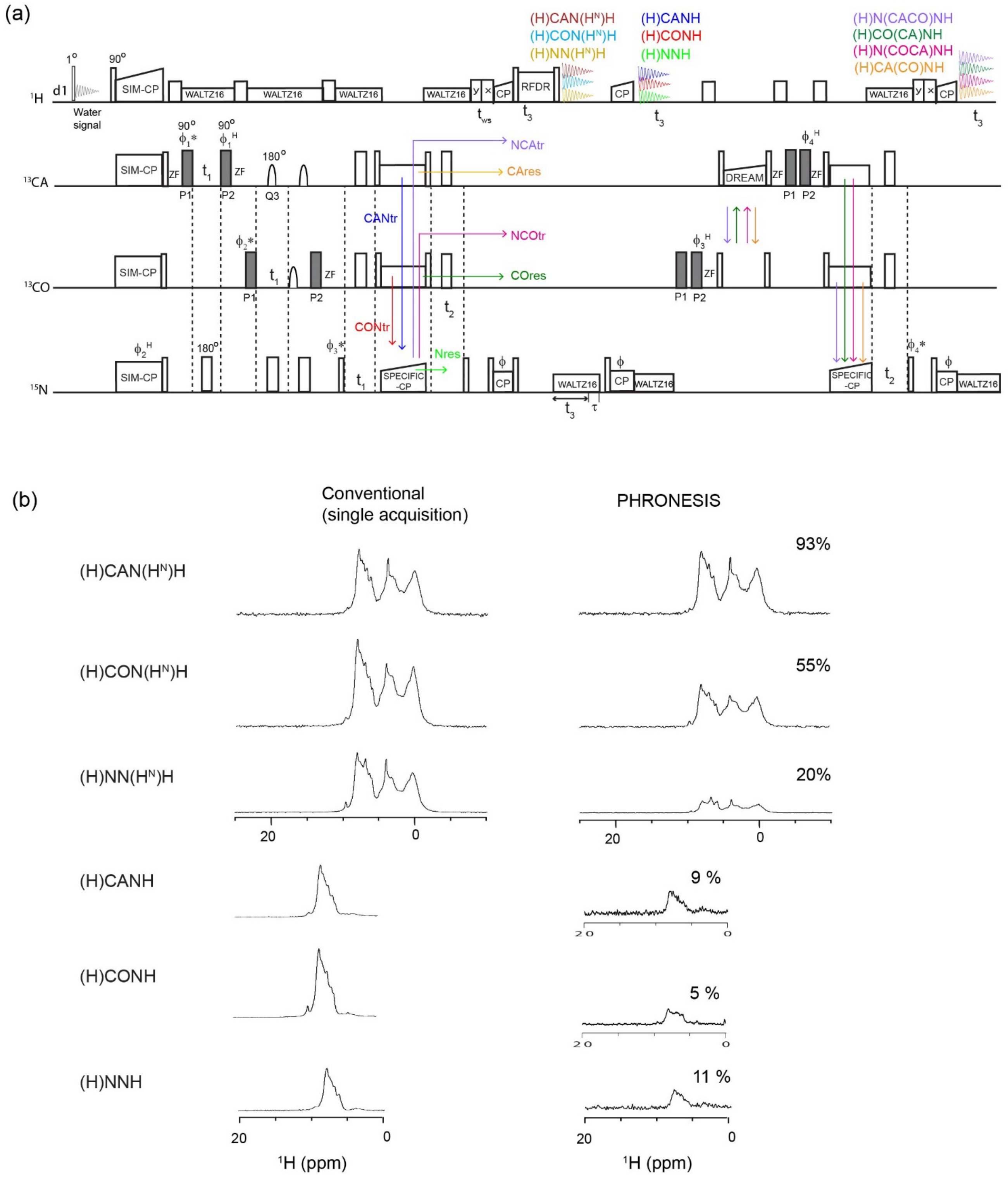

Figure S14: (a) Modified PHRONESIS experiment obtained by switching the 2nd and 3rd acquisition periods of Figure 1a. High-intensity HH 1D spectra are obtained by placing the RFDR mixing before 2nd acquisition period. (b) Comparison of the conventional single-acquisition and PHRONESIS spectra obtained from the 1st increment (t_1=t_2=0). Note that the four spectra obtained in the 4th acquisition (not shown) are identical to Figure S10. Although the spectra were acquired with sixteen-step Hadamard phase cycling, the 2nd acquisition data sets (H)CANHH, (H)CONHH, and (H)NHH utilize only four-step phase cycling (ϕ_1^H and ϕ_2^H). Therefore, the pulse sequence can also be modified to acquire four different RFDR mixing periods by switching the mixing period after every four interleaved scans of Hadamard phase cycling.

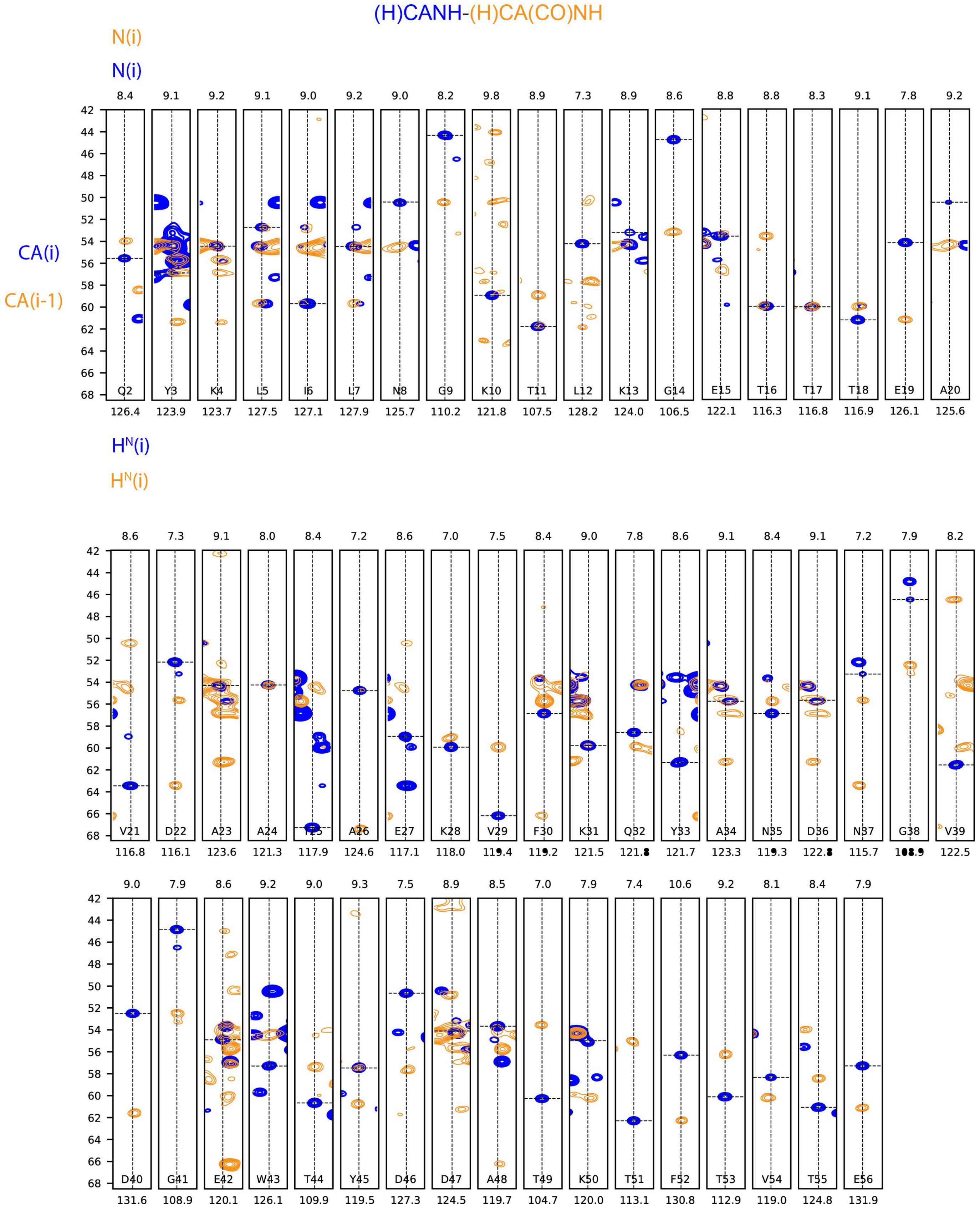

Figure S15: Sequential assignment of (H)CANH and (H)CA(CO)NH 3D spectra.

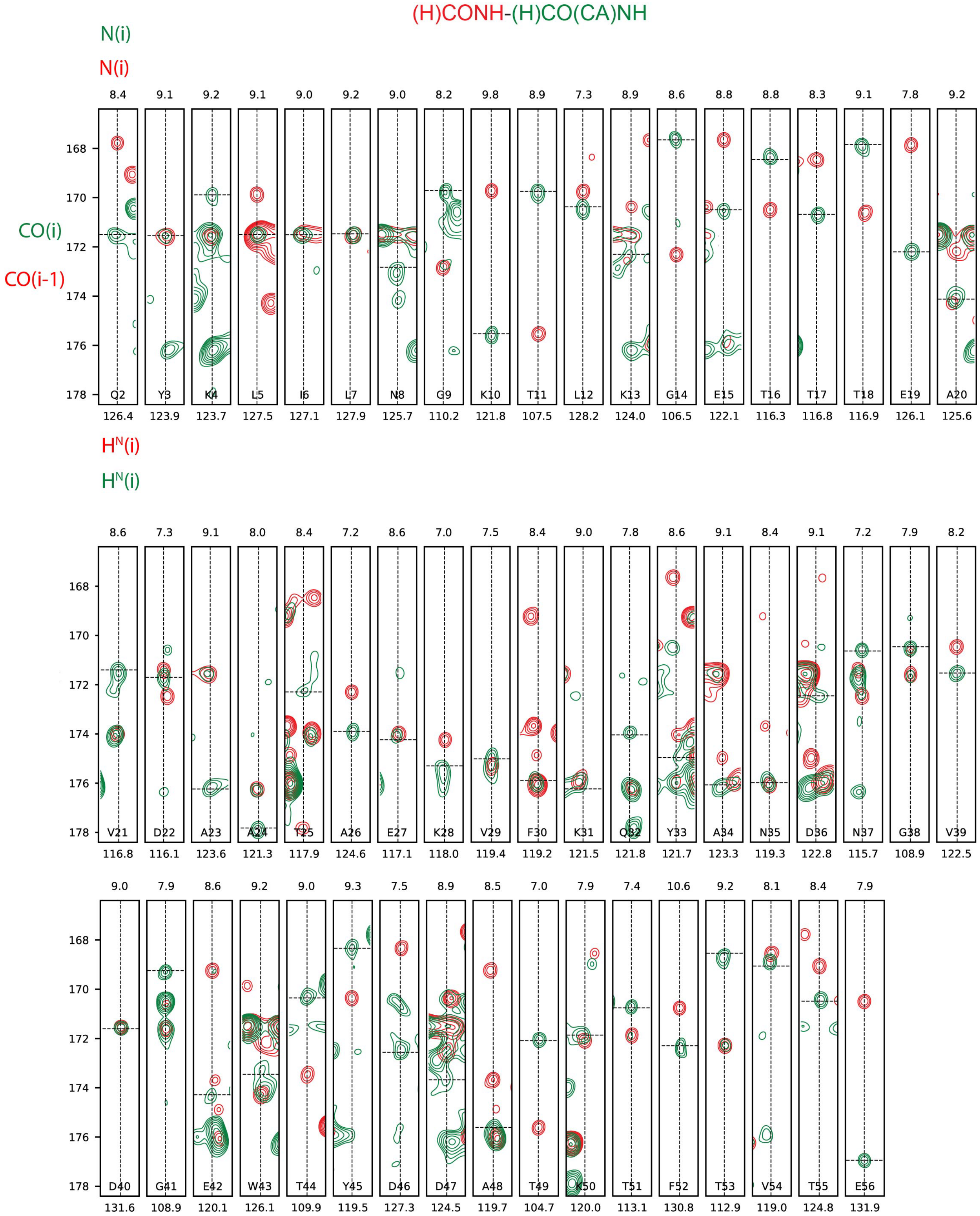

Figure S16: Sequential assignment of (H)CONH and (H)CO(CA)NH 3D spectra.

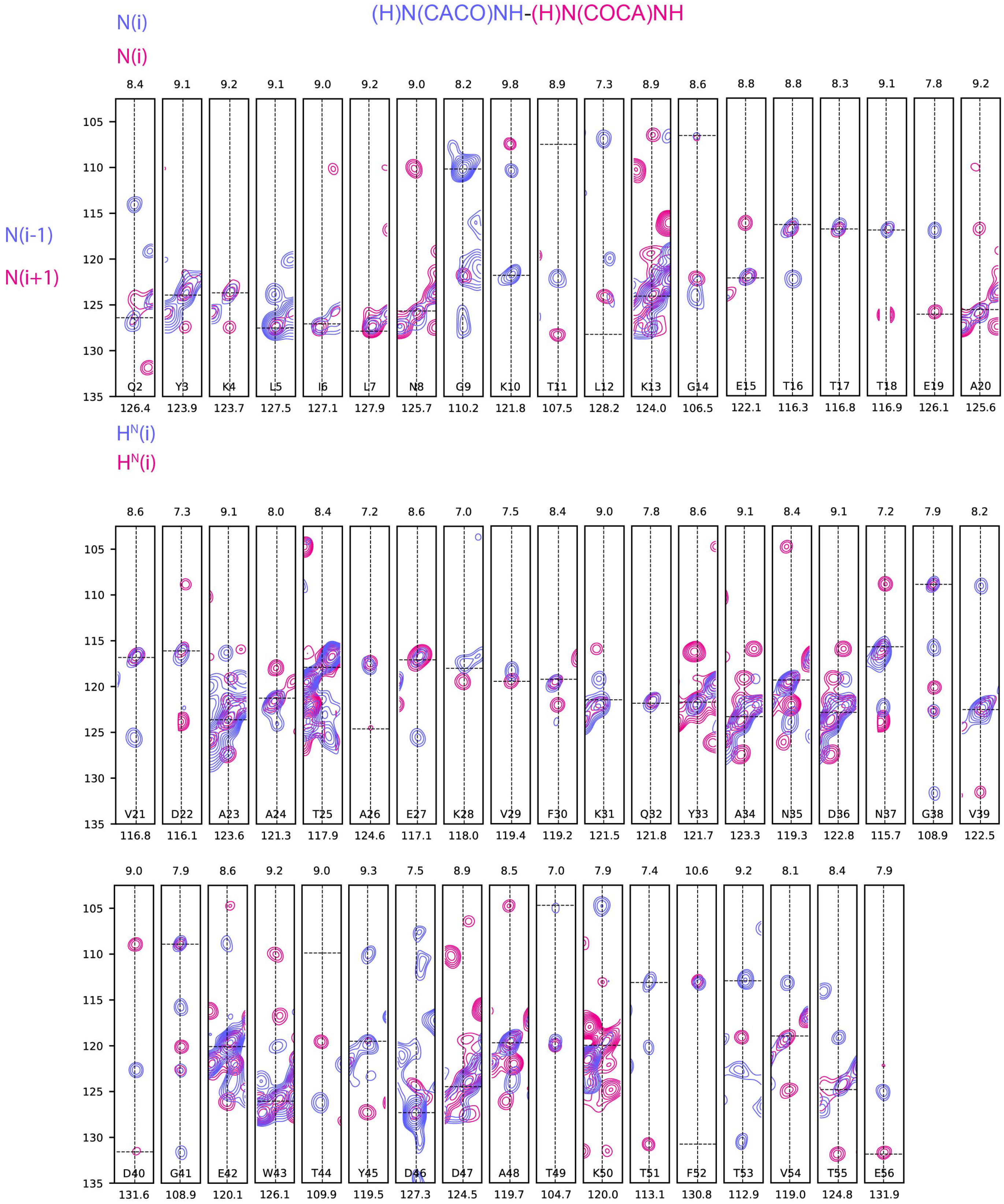

Figure S17: Sequential assignment of (H)N(COCA)NH and (H)N(CACO)NH 3D spectra.

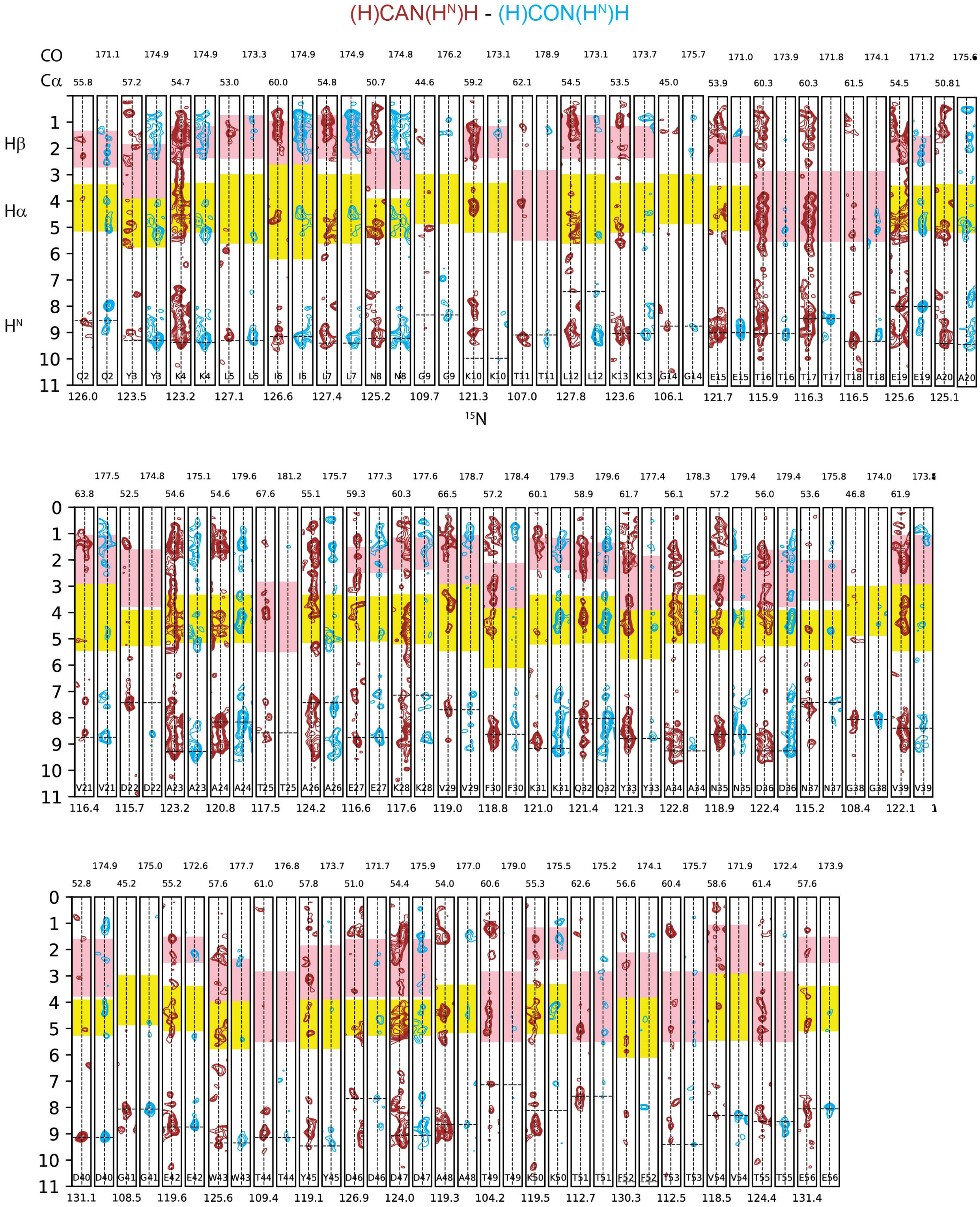

Figure S18: HA assignment (shaded in yellow) is obtained by analyzing the (H)CAN(H)H and (H)CONHH 3D spectra with corresponding N, CA, CO, and H^N assignments obtained from Figures S14-S16. The yellow and pink shaded regions represent expected HA and HB shifts calculated from the BMRB database. HB shifts were not analyzed due to low resolution and low signal intensities.

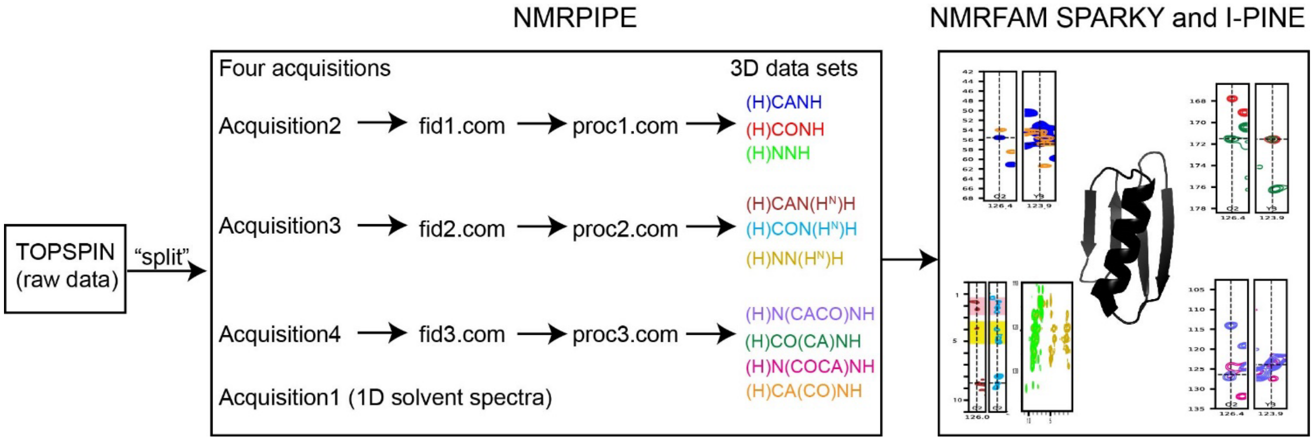

Figure S19: Protocol for 3D PHRONESIS data processing and sequential protein assignment. Pulse sequence and processing scripts (fid.com and proc.com) are given below.

3D PHRONESIS pulse sequence for BRUKER Avance NEO console operated with Topspin-4.0.9:

```
#include <Avance.incl>
#include <De.incl>

define loopcounter count
define loopcounter count1
define loopcounter count2
"count=td2/128"
"count1=td1/2"
"in0=inf2/2"
"in10=inf1/2"
"d0=0.1u"
"d10=0.1u"
"count2=(count/2)"
"in20=inf2"
"l20=0"
"d20=0.1u"

"l11=0"
"acqt0=-p1/2"

1 ze
  "cnst21=0"
2 40u do:f3
  40u do:f1
  40u do:f2
  d1 st0                                  ; Recycle delay
 1m

if "l20==count2"{
"l20=0"
"d20=0.1u"
}

 10u pl5:f3
 10u pl7:f2
 10u pl1:f1

"cnst23=-d0*2*360*46*150.868-l11*90"     ; t1 dependent phase modulation moves the  t1 offset to CA (54 ppm); 1 ppm = 150.868 Hz
 1u ip13+cnst23                          ; i.e from o2p to (o2p-46) = (100-46) = 54 ppm

 "cnst24=d20*2*360*68*150.868+l11*90"    ; t1 dependent phase modulation moves the  t1 offset to CO (175 ppm), 1 ppm = 150.868 Hz
1u ip14+cnst24                           ; i.e from o2p to (o2p+68) = (100+68) = 168 ppm

(p35 pl35 ph3):f1                        ; 1° 1H pulse

goscnp ph29                              ;******* 1st acquisition, water or solvent signal  *******

1m st

1u fq=cnst13(bf ppm):f2                                  ;cnst13=125 ppm
(p1 pl1 ph1):f1                                          ;1H 90° pulse
(p15:sp1 ph2):f1   (p15 pl7 ph6):f2   (p15 pl5 ph17):f3  ; Broadband SIM-CP
(p2 pl2 ph3):f2 (p3 pl3 ph3):f3                          ;13C and 15N 90° pulses
1u fq=cnst11(bf ppm):f2                                  ;cnst11=100 ppm

(p14 pl14 ph2):f1                                        ;1H HORROR pulse

.6u pl12:f1
.6u cpd1:f1                                              ; WALTZ 1H decoupling
(p36:sp36 ph13):f2                                       ; GENETICS-AI pulse for CA excitation
                                                         ;t1 evolution for CA
d0
(p3*2 pl3 ph2):f3                                        ;15N 180° pulse
d0
(p37:sp37 ph7):f2                                        ; GENETICS-AI flipback pulse for CA
1u do:f1
(p14 pl14 ph2):f1                                        ;1H HORROR pulse

.6u pl12:f1
.6u cpd3:f1                                              ; WALTZ 1H decoupling
(p38:sp38 ph14):f2                                       ; GENETICS-AI pulse for CO excitation
                                                         ;t1 evolution for CO
d20
(center (p3*2 pl3 ph2):f3 (p12:sp12 ph2):f2)             ; 180° pulse on N and CA
d20
```

```
10u
(p13:sp13 ph2):f2                                   ;180O CO refocusing pulse
10u
(center (p3*2 pl3 ph2):f3 (p12:sp12 ph2):f2)        ; 180O pulse on N and CA
(p39:sp39 ph2):f2                                   ; GENETICS-AI  flipback pulse for CO
1u do:f1
(p14 pl14 ph2):f1                                   ;1H HORROR pulse

.6u pl12:f1
.6u cpd2:f1                                         ;1H WALTZ decoupling
(p3 pl3 ph8):f3                                     ;15N 90º pulse
                                                    ;15N t1 evolution
d20
(p2*2 pl2 ph2):f2                                   ;Hard 180º pulse on 13C
d20
(p2 pl2 ph3):f2                                     ;13C 90º pulse
1u do:f1


1u fq=cnst10(bf ppm):f2                             ;cnst10=115 ppm
(p19 pl8 ph2):f2 (p19:sp4 ph2):f3                   ;Broadband SPECIFIC-CP; CAN, CON, NCA, and NCO tranfer
1u fq=cnst11(bf ppm):f2                             ;cnst11=100 ppm
(p2 pl2 ph5):f2                                     ;13C 90º pulse

.6u pl12:f1 ;
.6u cpd4:f1 ;                                       ;1H WALTZ decoupling
                                                    ;15N t2 evolution
d10
(p2*2 pl2 ph2):f2                                   ;13C 180º pulse
d10
1u do:f1
(p3 pl3 ph11):f3                                    ;15N 90º pulse
                                                    ;Water suppression (MISSISSIPPI)
10u pl6:f1                                          ;30 kHz d11 = 100 ms
4u cw:f1 ph19
d11*0.25
4u cw:f1 ph15
d11*0.25
4u cw:f1 ph19
d11*0.25
4u cw:f1 ph15
d11*0.25
4u do:f1

(p3 pl3 ph3):f3                                     ;15N 90º pulse
(p18 pl15 ph4):f3 (p18:sp2 ph2):f1                  ;NH CP
(p3 pl3 ph3):f3                                     ;15N 90º pulse

4u pl30:f3           ; WALTZ16 decoupling on 15N
1u cpd5:f3
goscnp ph31          ;******* 2nd 1H acquisition (H)CANH, (H)CONH, and (H)NNH  *******
1270u                ; Additional WALTZ16 delay on 15N (τ in Figure 1) to align the residual 15N polarization along z-direction
1u do:f3

1m st

(p3 pl3 ph5):f3                                     ;15N 90º pulse
(p18 pl15 ph4):f3 (p18:sp2 ph2):f1                  ;NH CP

(p1 pl1 ph3):f1                                     ;1H 90º pulse
5 d13                                               ;HH RFDR
 (p1*2 pl1 ph15):f1
 d12
 (p1*2 pl1 ph19):f1
 d12
 (p1*2 pl1 ph15):f1
 d12
 (p1*2 pl1 ph19):f1
 d12
 (p1*2 pl1 ph15):f1
 d12
 (p1*2 pl1 ph19):f1
 d13             ;6.44
lo to 5 times l5
(p1 pl1 ph5):f1                                     ;1H 90º pulse

1u pl30:f3
1u cpd5:f3
```

```
goscnp ph31                  ;******* 3rd 1H acquisition (H)CAN(H)H, (H)CON(H)H, and (H)NN(H)H*******
1u do:f3

1m st

(p38:sp38 ph2):f2                              ; GENETICS-AI  pulse for CO excitation
(p39:sp39 ph16):f2                             ; GENETICS-AI  pulse for CO flipback
(p14 pl14 ph2):f1

(p2 pl2 ph3):f2                                ;13C 90º pulse
1u fq=cnst14(bf ppm):f2                        ;cnst14=57 ppm
(p21:sp6 ph2):f2                               ; Bi-directional DREAM; CACO and COCA transfer
1u fq=cnst11(bf ppm):f2                        ;cnst11=100 ppm
(p2 pl2 ph5):f2                                ;13C 90º pulse
(p14 pl14 ph2):f1                              ;1H HORROR pulse

(p36:sp36 ph2):f2                              ; GENETICS-AI pulse for CA excitation
(p37:sp37 ph21):f2                             ; GENETICS-AI pulse for CA flipback
(p14 pl14 ph2):f1                              ;1H HORROR pulse

(p2 pl2 ph3):f2                                ;13C 90º pulse
                                               ;Broadband SPECIFIC-CP; NCA and NCO transfer ;
1u fq=cnst10(bf ppm):f2                        ;cnst10=115 ppm
(p19 pl8 ph2):f2 (p19:sp4 ph2):f3
1u fq=cnst11(bf ppm):f2                        ;cnst11=100 ppm

.6u pl12:f1 ;                                  ;15N t2 evolution
.6u cpd6:f1 ;
d10
(p2*2 pl2 ph2):f2                              ;13C 180º pulse
d10
1u do:f1
(p3 pl3 ph11):f3                               ;15N 90º pulse
                                               ;Water suppression
10u pl6:f1
4u cw:f1 ph19
d11*0.125
4u cw:f1 ph15
d11*0.125
4u do:f1

(p3 pl3 ph3):f3                                ;15N 90º pulse
(p18 pl15 ph4):f3 (p18:sp2 ph2):f1             ;NH CP

1u pl30:f3
1u cpd7:f3
goscnp ph31                ;******* 4th acquisition  (H)CA(CO)NH, (H)CO(CA)NH, (H)N(CACO)NH, and (H)N(COCA)NH  *******
1u do:f3

1m
1m ipp4 ipp31
lo to 2 times ns
1m rpp4 rpp31
10m wr #0  if #0 zd
                                          ;Four loops for Hadamrd phase encoding
1m ip7*2
lo to 2 times 2
1m rp7
1m ip17*2
lo to 2 times 2
1m rp17
1m ip16*2
lo to 2 times 2
1m rp16
1m ip21*2
lo to 2 times 2
1m rp21
                                          ;t1 and t2 loops with States-TPPI  phase switching
1m ip8
1m iu11
lo to 2 times 2
1m id0
1m id20
1m iu20
lo to 2 times count
1m rd0
1m rd20
```

```
1m ip11
1m ip12
lo to 2 times 2
1u id10
lo to 2 times count1
HaltAcqu, 1m
exit

ph1= 1
ph2 = 0
ph3 = 3
ph4 = 0 2
ph5 = 1
ph6 = 2
ph7 = 0
ph17 = 0
ph16 = 0
ph21 = 0
ph13 = 0
ph14 = 0
ph8 = 1
ph9 = 0
ph10 = 0
ph11 = 1
ph12 = 1
ph19 = 1
ph15 = 0
ph18 = 1
ph29 = 0                ;Receiver phase for 1st acquisition (solvent signal)
ph31 = 0 2              ;Receiver phase for 2nd 3rd and 4th acquisitions
```

NMRPIPE script “fid1.com“:

```
#!/bin/csh
bruk2pipe -in ./ser \
  -bad 0.0 -ext -aswap -AMX -decim 200 -dspfvs 21 -grpdly 76  \
  -xN          1792  -yN          1152  -zN   36  \
  -xT           832  -yT           576  -zT    18 \ # 16 step Hadamard encoding, 36 CA t1 (16*36=576), and 18 15N t2 complex points
  -xMODE        DQD  -yMODE     Complex  -zMODE      Complex  \
  -xSW   100000.000  -ySW       4444.44  -zSW        2222.22  \
  -xOBS     599.975  -yOBS      150.871  -zOBS        60.802  \
  -xCAR       4.282  -yCAR         54.1   -zCAR       118.7 \
  -xLAB          HN  -yLAB          13C   -zLAB          15N  \
  -ndim           3  -aq2D      Complex                    \
  -out ./hCANH/test%03d.fid -verb -ov
sleep 5

bruk2pipe -in ./ser \
  -bad 0.0 -ext -aswap -AMX -decim 200 -dspfvs 21 -grpdly 76  \
  -xN        1792  -yN   1152  -zN   36  \
  -xT         832  -yT    576  -zT   18  \  # 16 step Hadamard encoding, two sets of 18 CO t1 (16*2*18=576), and 18 15N t2 complex points
  -xMODE      DQD  -yMODE    Complex  -zMODE      Complex  \
  -xSW   100000.000  -ySW       2222.22  -zSW        2222.22  \
  -xOBS     599.975  -yOBS      150.871  -zOBS        60.802  \
  -xCAR       4.282  -yCAR        167.5     -zCAR       118.7  \
  -xLAB          HN  -yLAB          13C  -zLAB          15N  \
  -ndim           3  -aq2D      Complex                   \
  -out ./hCONH/test%03d.fid -verb -ov
sleep 5

bruk2pipe -in ./ser \
  -bad 0.0 -ext -aswap -AMX -decim 200 -dspfvs 21 -grpdly 76  \
  -xN        1792  -yN          1152  -zN      36  \
  -xT         832  -yT     576  -zT     \  # 16 step Hadamard encoding, two sets of 18 15N t1 (16*2*18=576), and 18 15N t2 complex points
  -xMODE      DQD  -yMODE    Complex  -zMODE      Complex  \
  -xSW   100000.000  -ySW       2222.22  -zSW        2222.22  \
  -xOBS     599.975  -yOBS       60.802  -zOBS        60.802  \
  -xCAR       4.282  -yCAR        118.7     -zCAR       118.7  \
```

```
  -xLAB              HN  -yLAB             15N  -zLAB             15N  \
  -ndim               3  -aq2D         Complex                       \
  -out ./hNNH/test%03d.fid -verb -ov
sleep 5
```

NMRPIPE script “proc1.com“:

```
#!/bin/csh
xyz2pipe -in hCANH/test%03d.fid -x -verb                  \
| nmrPipe  -fn COADD -axis Y -cList 1 -1 1 -1 1 -1 1 -1 1 -1 1 -1 1 -1 1 -1 -time  \   # 2nd column of Figure S8
| nmrPipe  -fn SP -off 0.5 -end 1.0  \
| nmrPipe  -fn ZF -size 32000                               \
| nmrPipe  -fn FT                                  \
| nmrPipe  -fn PS -p0 128.0 -p1 0.0 -di                  \
| nmrPipe  -fn EXT -x1 5.5ppm -xn 11.5ppm -sw \
| nmrPipe  -fn TP                                  \
| nmrPipe  -fn SP -off 0.5 -end 1.0  \
| nmrPipe  -fn ZF  -size 320                             \
| nmrPipe  -fn FT -alt -neg                                \
| nmrPipe  -fn PS -p0 0.0 -p1 0.0 -di                    \
| pipe2xyz -out F3F1-t2/test%03d.ft2 -y

xyz2pipe -in F3F1-t2/test%03d.ft2 -z -verb                   \
| nmrPipe  -fn SP -off 0.5 -end 1.0  \
| nmrPipe  -fn ZF -size 320                              \
| nmrPipe  -fn FT -alt -neg                                  \
| nmrPipe  -fn PS -p0 180.0 -p1 0.0 -di                \
| pipe2xyz -out F3F2-F1/test%03d.ft3 -z

xyz2pipe -in F3F2-F1/test%03d.ft3 -x > hCANH.ft          # 3D spectrum in “nmrpipe“ format
pipe2ucsf hCANH.ft hCANH.ucsf                                # 3D spectrum in “sparky“ format
#################################################################
xyz2pipe -in hCONH/test%03d.fid -x -verb                  \
| nmrPipe  -fn COADD -axis Y -cList 1 1 1 1 1 1 1 1 1 1 1 1 1 1 1 1 -time  \  # 1st column of Figure S8
| nmrPipe  -fn SP -off 0.5 -end 1.0  \
| nmrPipe  -fn ZF -size 32000                               \
| nmrPipe  -fn FT                                  \
| nmrPipe  -fn PS -p0 128.0 -p1 0.0 -di                  \
| nmrPipe  -fn EXT -x1 5.5ppm -xn 11.5ppm -sw \
| nmrPipe  -fn TP                                  \
| nmrPipe  -fn EXT -x1 1 -xn 18 -sw                   \           # 1 to 18 t1 increments
| nmrPipe  -fn SP -off 0.5 -end 1.0  \
| nmrPipe  -fn ZF -size 320                              \
| nmrPipe  -fn FT -alt                                 \
| nmrPipe  -fn PS -p0 0.0 -p1 0.0 -di                    \
| pipe2xyz -out xy1/test%03d.ft2 -y

xyz2pipe -in fid1/test%03d.fid -x -verb                  \
| nmrPipe  -fn COADD -axis Y -cList 1 1 1 1 1 1 1 1 1 1 1 1 1 1 1 1 -time  \ # 1st column of Figure S8
| nmrPipe  -fn SP -off 0.5 -end 1.0  \
| nmrPipe  -fn ZF -size 32000                               \
| nmrPipe  -fn FT                                  \
| nmrPipe  -fn PS -p0 128.0 -p1 0.0 -di                  \
| nmrPipe  -fn EXT -x1 5.5ppm -xn 11.5ppm -sw \
| nmrPipe  -fn TP                                  \
| nmrPipe  -fn EXT -x1 19 -xn 36 -sw                  \   # 19 to 36 t1 increments
| nmrPipe  -fn SP -off 0.5 -end 1.0  \
| nmrPipe  -fn ZF -size 320                              \
| nmrPipe  -fn FT -alt                                 \
| nmrPipe  -fn PS -p0 0.0 -p1 0.0 -di                    \
| pipe2xyz -out xy2/test%03d.ft2 -y

addNMR -in1 xy1/test%03d.ft2 -in2 xy2/test%03d.ft2 -out  F3F1-t2/test%03d.ft2 –add # sum of two CO t1 data sets

xyz2pipe -in  F3F1-t2/test%03d.ft2 -z -verb                   \
| nmrPipe  -fn SP -off 0.5 -end 1.0  \
| nmrPipe  -fn ZF -size 320                              \
| nmrPipe  -fn FT -alt -neg                                  \
| nmrPipe  -fn PS -p0 180.0 -p1 0.0 -di                \
| pipe2xyz -out F3F2-F1/test%03d.ft3 -z

xyz2pipe -in F3F2-F1/test%03d.ft3 -x > hCONH.ft
pipe2ucsf hCONH.ft hCONH.ucsf
######################################################################
xyz2pipe -in hNNH/test%03d.fid -x -verb                  \
| nmrPipe  -fn COADD -axis Y -cList 1 1 -1 -1 1 1 -1 -1 1 1 -1 -1 1 1 -1 -1 -time  \  # 3rd column of Figure S8
| nmrPipe  -fn SP -off 0.5 -end 1.0  \
| nmrPipe  -fn ZF -size 32000                               \
```

```
| nmrPipe  -fn FT                                             \
| nmrPipe  -fn PS -p0 -52.0 -p1 0.0 -di                   \
| nmrPipe  -fn EXT -x1 5.5ppm -xn 11.5ppm -sw \
| nmrPipe  -fn TP                                             \
| nmrPipe  -fn EXT -x1 1 -xn 18 -sw                   \  #1 to 18 t1 increments
| nmrPipe  -fn SP -off 0.5 -end 1.0  \
| nmrPipe  -fn ZF -size 320                               \
| nmrPipe  -fn FT -alt                                    \
| nmrPipe  -fn PS -p0 0.0 -p1 0.0 -di                     \
| pipe2xyz -out xy1/test%03d.ft2 -y

xyz2pipe -in fid2/test%03d.fid -x -verb                  \
| nmrPipe  -fn COADD -axis Y -cList 1 1 -1 -1 1 1 -1 -1 1 1 -1 -1 1 1 -1 -1 -time  \  # 3rd column of Figure S8
| nmrPipe  -fn SP -off 0.5 -end 1.0  \
| nmrPipe  -fn ZF -size 32000                             \
| nmrPipe  -fn FT                                             \
| nmrPipe  -fn PS -p0 -52.0 -p1 0.0 -di                   \
| nmrPipe  -fn EXT -x1 5.5ppm -xn 11.5ppm -sw \
| nmrPipe  -fn TP                                             \
| nmrPipe  -fn EXT -x1 19 -xn 36 -sw                   \  #19 to 36 t1 increments
| nmrPipe  -fn SP -off 0.5 -end 1.0  \
| nmrPipe  -fn ZF -size 320                               \
| nmrPipe  -fn FT -alt                                    \
| nmrPipe  -fn PS -p0 0.0 -p1 0.0 -di                     \
| pipe2xyz -out xy2/test%03d.ft2 -y

addNMR -in1 xy1/test%03d.ft2 -in2 xy2/test%03d.ft2 -out  F3F1-t2/test%03d.ft2 –add # sum of two 15N t1 data sets

xyz2pipe -in  F3F1-t2/test%03d.ft2 -z -verb                    \
| nmrPipe  -fn SP -off 0.5 -end 1.0  \
| nmrPipe  -fn ZF -size 320                               \
| nmrPipe  -fn FT -alt -neg                                   \
| nmrPipe  -fn PS -p0 180.0 -p1 0.0 -di                \
| pipe2xyz -out F3F2-F1/test%03d.ft3 -z

xyz2pipe -in F3F2-F1/test%03d.ft3 -x > hNNH.ft
pipe2ucsf hNNH.ft hNNH.ucsf
```

NMRPIPE script “fid2.com“:

```
#!/bin/csh
bruk2pipe -in ./ser \
  -bad 0.0 -ext -aswap -AMX -decim 200 -dspfvs 21 -grpdly 76  \
  -xN              1792  -yN               1152  -zN               36  \
  -xT               832  -yT                576  -zT                18  \
  -xMODE            DQD  -yMODE        Complex  -zMODE        Complex  \
  -xSW       100000.000  -ySW         4444.44  -zSW         2222.22  \
  -xOBS         599.975  -yOBS        150.871  -zOBS          60.802  \
  -xCAR           4.282  -yCAR          54.1   -zCAR         118.7 \
  -xLAB              HN  -yLAB            13C   -zLAB             15N  \
  -ndim               3  -aq2D         Complex                        \
  -out ./hCANHH/test%03d.fid -verb -ov

sleep 5


bruk2pipe -in ./ser \
  -bad 0.0 -ext -aswap -AMX -decim 200 -dspfvs 21 -grpdly 76  \
  -xN              1792  -yN              1152  -zN               36  \
  -xT               832  -yT               576  -zT                18  \
  -xMODE            DQD  -yMODE        Complex  -zMODE        Complex  \
  -xSW       100000.000  -ySW         2222.22  -zSW         2222.22  \
  -xOBS         599.975  -yOBS         150.871  -zOBS          60.802  \
  -xCAR           4.282  -yCAR          167.5     -zCAR           118.7  \
  -xLAB              HN  -yLAB             13C  -zLAB             15N  \
  -ndim               3  -aq2D         Complex                        \
  -out ./hCONHH/test%03d.fid -verb -ov
sleep 5

bruk2pipe -in ./ser \
  -bad 0.0 -ext -aswap -AMX -decim 200 -dspfvs 21 -grpdly 76  \
  -xN              1792  -yN              1152  -zN               36  \
  -xT               832  -yT               576  -zT                18  \
  -xMODE            DQD  -yMODE        Complex  -zMODE        Complex  \
  -xSW       100000.000  -ySW         2222.22  -zSW         2222.22  \
  -xOBS         599.975  -yOBS         60.802  -zOBS          60.802  \
  -xCAR           4.282  -yCAR          118.7     -zCAR           118.7  \
  -xLAB              HN  -yLAB             15N  -zLAB             15N  \
```

```
  -ndim              3  -aq2D        Complex                    \
  -out ./hNNHH/test%03d.fid -verb -ov
sleep 5
```

NMRPIPE script “proc2.com“:

```
#!/bin/csh
xyz2pipe -in hCANHH/test%03d.fid -x -verb                \
| nmrPipe  -fn COADD -axis Y -cList 1 -1 1 -1 1 -1 1 -1 1 -1 1 -1 1 -1 1 -1 -time  \  # 2nd column of Figure S8
| nmrPipe -fn EM -lb 100.0              \
| nmrPipe  -fn ZF -size 32000                          \
| nmrPipe  -fn FT                                \
| nmrPipe  -fn PS -p0 128.0 -p1 0.0 -di                \
| nmrPipe  -fn EXT -x1 0ppm -xn 11.5ppm -sw \
| nmrPipe  -fn TP                                \
| nmrPipe  -fn SP -off 0.5 -end 1.0  \
| nmrPipe  -fn ZF  -size 320                        \
| nmrPipe  -fn FT -alt -neg                          \
| nmrPipe  -fn PS -p0 0.0 -p1 0.0 -di                  \
| pipe2xyz -out F3F1-t2/test%03d.ft2 -y

xyz2pipe -in F3F1-t2/test%03d.ft2 -z -verb                 \
| nmrPipe  -fn SP -off 0.5 -end 1.0  \
| nmrPipe  -fn ZF -size 320                         \
| nmrPipe  -fn FT -alt -neg                            \
| nmrPipe  -fn PS -p0 180.0 -p1 0.0 -di              \
| pipe2xyz -out F3F2-F1/test%03d.ft3 -z

xyz2pipe -in F3F2-F1/test%03d.ft3 -x > hCANHH.ft
pipe2ucsf hCANHH.ft hCANHH.ucsf
################################
xyz2pipe -in hCONHH/test%03d.fid -x -verb                \
| nmrPipe  -fn COADD -axis Y -cList 1 1 1 1 1 1 1 1 1 1 1 1 1 1 1 1 -time  \ # 1st column of Figure S8
| nmrPipe -fn EM -lb 100.0              \
| nmrPipe  -fn ZF -size 32000                          \
| nmrPipe  -fn FT                                \
| nmrPipe  -fn PS -p0 128.0 -p1 0.0 -di                \
| nmrPipe  -fn EXT -x1 0ppm -xn 11.5ppm -sw \
| nmrPipe  -fn TP                                \
| nmrPipe  -fn EXT -x1 1 -xn 18 -sw                 \
| nmrPipe  -fn SP -off 0.5 -end 1.0  \
| nmrPipe  -fn ZF -size 320                         \
| nmrPipe  -fn FT -alt                           \
| nmrPipe  -fn PS -p0 0.0 -p1 0.0 -di                  \
| pipe2xyz -out xy1/test%03d.ft2 -y

xyz2pipe -in fid1/test%03d.fid -x -verb                \
| nmrPipe  -fn COADD -axis Y -cList 1 1 1 1 1 1 1 1 1 1 1 1 1 1 1 1 -time  \
| nmrPipe -fn EM -lb 100.0              \
| nmrPipe  -fn ZF -size 32000                          \
| nmrPipe  -fn FT                                \
| nmrPipe  -fn PS -p0 128.0 -p1 0.0 -di                \
| nmrPipe  -fn EXT -x1 0ppm -xn 11.5ppm -sw \
| nmrPipe  -fn TP                                \
| nmrPipe  -fn EXT -x1 19 -xn 36 -sw                 \
| nmrPipe  -fn SP -off 0.5 -end 1.0  \
| nmrPipe  -fn ZF -size 320                         \
| nmrPipe  -fn FT -alt                           \
| nmrPipe  -fn PS -p0 0.0 -p1 0.0 -di                  \
| pipe2xyz -out xy2/test%03d.ft2 -y

addNMR -in1 xy1/test%03d.ft2 -in2 xy2/test%03d.ft2 -out  F3F1-t2/test%03d.ft2 –add # sum of two CO t1 data sets

xyz2pipe -in  F3F1-t2/test%03d.ft2 -z -verb                 \
| nmrPipe  -fn SP -off 0.5 -end 1.0  \
| nmrPipe  -fn ZF -size 320                         \
| nmrPipe  -fn FT -alt -neg                            \
| nmrPipe  -fn PS -p0 180.0 -p1 0.0 -di              \
| pipe2xyz -out F3F2-F1/test%03d.ft3 -z

xyz2pipe -in F3F2-F1/test%03d.ft3 -x > hCONHH.ft
pipe2ucsf hCONHH.ft hCONHH.ucsf
###############################
xyz2pipe -in hNNHH/test%03d.fid -x -verb                \
| nmrPipe  -fn COADD -axis Y -cList 1 1 -1 -1 1 1 -1 -1 1 1 -1 -1 1 1 -1 -1 -time  \ # 3rd column of Figure S8
| nmrPipe  -fn SP -off 0.5 -end 1.0  \
| nmrPipe  -fn ZF -size 32000                          \
| nmrPipe  -fn FT                                \
| nmrPipe  -fn PS -p0 -52.0 -p1 0.0 -di                \
```

```
| nmrPipe  -fn EXT -x1 0ppm -xn 11.5ppm -sw \
| nmrPipe  -fn TP                                        \
| nmrPipe  -fn EXT -x1 1 -xn 18 -sw                  \
| nmrPipe  -fn SP -off 0.5 -end 1.0  \
| nmrPipe  -fn ZF -size 320                          \
| nmrPipe  -fn FT -alt                               \
| nmrPipe  -fn PS -p0 0.0 -p1 0.0 -di                \
| pipe2xyz -out xy1/test%03d.ft2 -y


xyz2pipe -in fid2/test%03d.fid -x -verb               \
| nmrPipe  -fn COADD -axis Y -cList 1 1 -1 -1 1 1 -1 -1 1 1 -1 -1 1 1 -1 -1 -time  \
| nmrPipe  -fn SP -off 0.5 -end 1.0  \
| nmrPipe  -fn ZF -size 32000                           \
| nmrPipe  -fn FT                                        \
| nmrPipe  -fn PS -p0 -52.0 -p1 0.0 -di                 \
| nmrPipe  -fn EXT -x1 0ppm -xn 11.5ppm -sw \
| nmrPipe  -fn TP                                        \
| nmrPipe  -fn EXT -x1 19 -xn 36 -sw                 \
| nmrPipe  -fn SP -off 0.5 -end 1.0  \
| nmrPipe  -fn ZF -size 320                          \
| nmrPipe  -fn FT -alt                               \
| nmrPipe  -fn PS -p0 0.0 -p1 0.0 -di                \
| pipe2xyz -out xy2/test%03d.ft2 -y

addNMR -in1 xy1/test%03d.ft2 -in2 xy2/test%03d.ft2 -out  F3F1-t2/test%03d.ft2 –add # sum of two 15N t1 data sets

xyz2pipe -in  F3F1-t2/test%03d.ft2 -z -verb                \
| nmrPipe  -fn SP -off 0.5 -end 1.0  \
| nmrPipe  -fn ZF -size 320                          \
| nmrPipe  -fn FT -alt -neg                              \
| nmrPipe  -fn PS -p0 180.0 -p1 0.0 -di              \
| pipe2xyz -out F3F2-F1/test%03d.ft3 -z

xyz2pipe -in F3F2-F1/test%03d.ft3 -x > hNNHH.ft
pipe2ucsf hNNHH.ft hNNHH.ucsf
```

NMRPIPE script “fid3.com“:

```
#!/bin/csh
bruk2pipe -in ./ser \
  -bad 0.0 -ext -aswap -AMX -decim 200 -dspfvs 21 -grpdly 76  \
  -xN              1792  -yN              1152  -zN               36  \
  -xT               832  -yT               576  -zT               18  \
  -xMODE            DQD  -yMODE        Complex  -zMODE        Complex  \
  -xSW       100000.000  -ySW         4444.44  -zSW         2222.22  \
  -xOBS         599.975  -yOBS        150.871  -zOBS          60.802  \
  -xCAR           4.282  -yCAR           54.1   -zCAR         118.7 \
  -xLAB              HN  -yLAB            13C   -zLAB            15N  \
  -ndim               3  -aq2D         Complex                      \
  -out ./hCAcoNH/test%03d.fid -verb -ov
sleep 5

bruk2pipe -in ./ser \
  -bad 0.0 -ext -aswap -AMX -decim 200 -dspfvs 21 -grpdly 76  \
  -xN              1792  -yN              1152  -zN               36  \
  -xT               832  -yT               576  -zT               18  \
  -xMODE            DQD  -yMODE        Complex  -zMODE        Complex  \
  -xSW       100000.000  -ySW         2222.22  -zSW         2222.22  \
  -xOBS         599.975  -yOBS        150.871  -zOBS          60.802  \
  -xCAR           4.282  -yCAR          167.5  -zCAR          118.7  \
  -xLAB              HN  -yLAB            13C  -zLAB            15N  \
  -ndim               3  -aq2D         Complex                      \
  -out ./hCOcaNH/test%03d.fid -verb -ov
sleep 5

bruk2pipe -in ./ser \
  -bad 0.0 -ext -aswap -AMX -decim 200 -dspfvs 21 -grpdly 76  \
  -xN              1792  -yN              1152  -zN               36  \
  -xT               832  -yT               576  -zT               18 \
  -xMODE            DQD  -yMODE        Complex  -zMODE        Complex  \
  -xSW       100000.000  -ySW         2222.22  -zSW         2222.22  \
  -xOBS         599.975  -yOBS         60.802  -zOBS          60.802  \
  -xCAR           4.282  -yCAR          118.7      -zCAR          118.7  \
  -xLAB              HN  -yLAB            15N  -zLAB            15N  \
  -ndim               3  -aq2D         Complex                      \
  -out ./hNcacoNH/test%03d.fid -verb –ov
sleep 5
```

```
bruk2pipe -in ./ser \
  -bad 0.0 -ext -aswap -AMX -decim 200 -dspfvs 21 -grpdly 76  \
  -xN              1792  -yN              1152  -zN                36  \
  -xT               832  -yT               576  -zT                18 \
  -xMODE            DQD  -yMODE        Complex  -zMODE        Complex  \
  -xSW       100000.000  -ySW         2222.22  -zSW        2222.22  \
  -xOBS         599.975  -yOBS         60.802  -zOBS          60.802  \
  -xCAR           4.282  -yCAR          118.7      -zCAR           118.7  \
  -xLAB              HN  -yLAB             15N  -zLAB             15N  \
  -ndim               3  -aq2D         Complex                       \
  -out ./hNcocaNH/test%03d.fid -verb –ov
```

NMRPIPE script “proc3.com“:

```
#!/bin/csh
xyz2pipe -in hCOcaNH/test%03d.fid -x -verb                  \
| nmrPipe  -fn COADD -axis Y -cList 1 1 1 1 -1 -1 -1 -1 -1 -1 -1 -1 1 1 1 1 -time  \ # 13th column of Figure S8
| nmrPipe  -fn SP -off 0.5 -end 1.0  \
| nmrPipe  -fn ZF -size 32000                              \
| nmrPipe  -fn FT                                  \
| nmrPipe  -fn PS -p0 134.0 -p1 0.0 -di                   \
| nmrPipe  -fn EXT -x1 5.5ppm -xn 11.5ppm -sw \
| nmrPipe  -fn TP                                  \
| nmrPipe  -fn EXT -x1 1 -xn 18 -sw                   \
| nmrPipe  -fn SP -off 0.5 -end 1.0  \
| nmrPipe  -fn ZF  -size 320                              \
| nmrPipe  -fn FT -alt                              \
| nmrPipe  -fn PS -p0 0.0 -p1 0.0 -di                     \
| pipe2xyz -out xy1/test%03d.ft2 -y

xyz2pipe -in fid1/test%03d.fid -x -verb                  \
| nmrPipe  -fn COADD -axis Y -cList 1 1 1 1 -1 -1 -1 -1 -1 -1 -1 -1 1 1 1 1 -time  \
| nmrPipe  -fn SP -off 0.5 -end 1.0  \
| nmrPipe  -fn ZF -size 32000                              \
| nmrPipe  -fn FT                                  \
| nmrPipe  -fn PS -p0 134.0 -p1 0.0 -di                   \
| nmrPipe  -fn EXT -x1 5.5ppm -xn 11.5ppm -sw \
| nmrPipe  -fn TP                                  \
| nmrPipe  -fn EXT -x1 19 -xn 36 -sw                   \
| nmrPipe  -fn SP -off 0.5 -end 1.0  \
| nmrPipe  -fn ZF  -size 320                              \
| nmrPipe  -fn FT -alt                              \
| nmrPipe  -fn PS -p0 0.0 -p1 0.0 -di                     \
| pipe2xyz -out xy2/test%03d.ft2 -y

addNMR -in1 xy1/test%03d.ft2 -in2 xy2/test%03d.ft2 -out  F3F1-t2/test%03d.ft2 –add # sum of two CO t1 data sets

xyz2pipe -in F3F1-t2/test%03d.ft2 -z -verb                   \
| nmrPipe  -fn SP -off 0.5 -end 1.0  \
| nmrPipe  -fn ZF -size 320                              \
| nmrPipe  -fn FT -alt -neg                                 \
| nmrPipe  -fn PS -p0 180.0 -p1 0.0 -di                \
| pipe2xyz -out F3F2-F1/test%03d.ft3 -z

xyz2pipe -in F3F2-F1/test%03d.ft3 -x > hCOcaNH.ft
pipe2ucsf hCOcaNH.ft hCOcaNH.ucsf
##############################
xyz2pipe -in hNcocaNH/test%03d.fid -x -verb                  \
| nmrPipe  -fn COADD -axis Y -cList 1 1 -1 -1 -1 -1 1 1 -1 -1 1 1 1 1 -1 -1 -time  \ # 15th column of Figure S8
| nmrPipe  -fn SP -off 0.5 -end 1.0  \
| nmrPipe  -fn ZF -size 32000                              \
| nmrPipe  -fn FT                                  \
| nmrPipe  -fn PS -p0 305.0 -p1 0.0 -di                   \
| nmrPipe  -fn EXT -x1 5.5ppm -xn 11.5ppm -sw \
| nmrPipe  -fn TP                                  \
| nmrPipe  -fn EXT -x1 1 -xn 18 -sw                   \
| nmrPipe  -fn SP -off 0.5 -end 1.0  \
| nmrPipe  -fn ZF  -size 320                              \
| nmrPipe  -fn FT -alt                              \
| nmrPipe  -fn PS -p0 0.0 -p1 0.0 -di                     \
| pipe2xyz -out xy1/test%03d.ft2 -y

xyz2pipe -in fid2/test%03d.fid -x -verb                  \
| nmrPipe  -fn COADD -axis Y -cList 1 1 -1 -1 -1 -1 1 1 -1 -1 1 1 1 1 -1 -1 -time  \
| nmrPipe  -fn SP -off 0.5 -end 1.0  \
| nmrPipe  -fn ZF -size 32000                              \
| nmrPipe  -fn FT                                  \
| nmrPipe  -fn PS -p0 305.0 -p1 0.0 -di                   \
```

```
| nmrPipe  -fn EXT -x1 5.5ppm -xn 11.5ppm -sw \
| nmrPipe  -fn TP                                              \
| nmrPipe  -fn EXT -x1 19 -xn 36 -sw                    \
| nmrPipe  -fn SP -off 0.5 -end 1.0  \
| nmrPipe  -fn ZF  -size 320                               \
| nmrPipe  -fn FT -alt                                       \
| nmrPipe  -fn PS -p0 0.0 -p1 0.0 -di                     \
| pipe2xyz -out xy2/test%03d.ft2 -y

addNMR -in1 xy1/test%03d.ft2 -in2 xy2/test%03d.ft2 -out  F3F1-t2/test%03d.ft2 –add # sum of two 15N t1 data sets

xyz2pipe -in F3F1-t2/test%03d.ft2 -z -verb                    \
| nmrPipe  -fn SP -off 0.5 -end 1.0  \
| nmrPipe  -fn ZF -size 320                                \
| nmrPipe  -fn FT -alt -neg                                    \
| nmrPipe  -fn PS -p0 0.0 -p1 0.0 -di                 \
| pipe2xyz -out F3F2-F1/test%03d.ft3 -z

xyz2pipe -in F3F2-F1/test%03d.ft3 -x > hNcocaNH.ft
pipe2ucsf hNcocaNH.ft hNcocaNH.ucsf
#####################################################
xyz2pipe -in hCAcoNH/test%03d.fid -x -verb                  \
| nmrPipe  -fn COADD -axis Y -cList 1 -1 1 -1 1 -1 1 -1 1 -1 1 -1 1 -1 1 -1 -time  \ # 2nd column of Figure S8
| nmrPipe  -fn SP -off 0.5 -end 1.0  \
| nmrPipe  -fn ZF -size 32000                                \
| nmrPipe  -fn FT                                              \
| nmrPipe  -fn PS -p0 134.0 -p1 0.0 -di                    \
| nmrPipe  -fn EXT -x1 5.5ppm -xn 11.5ppm -sw \
| nmrPipe  -fn TP                                              \
| nmrPipe  -fn SP -off 0.5 -end 1.0  \
| nmrPipe  -fn ZF  -size 320                               \
| nmrPipe  -fn FT -alt -neg                                  \
| nmrPipe  -fn PS -p0 0.0 -p1 0.0 -di                     \
| pipe2xyz -out F3F1-t2/test%03d.ft2 -y

xyz2pipe -in F3F1-t2/test%03d.ft2 -z -verb                    \
| nmrPipe  -fn SP -off 0.5 -end 1.0  \
| nmrPipe  -fn ZF -size 320                                \
| nmrPipe  -fn FT -alt -neg                                    \
| nmrPipe  -fn PS -p0 180.0 -p1 0.0 -di                 \
| pipe2xyz -out F3F2-F1/test%03d.ft3 -z

xyz2pipe -in F3F2-F1/test%03d.ft3 -x > hCAcoNH.ft
pipe2ucsf hCAcoNH.ft hCAcoNH.ucsf
###############################

xyz2pipe -in hNcacoNH/test%03d.fid -x -verb                  \
| nmrPipe  -fn COADD -axis Y -cList 1 1 -1 -1 1 1 -1 -1 1 1 -1 -1 1 1 -1 -1 -time  \ # 3rd column of Figure S8
| nmrPipe  -fn SP -off 0.5 -end 1.0  \
| nmrPipe  -fn ZF -size 32000                                \
| nmrPipe  -fn FT                                              \
| nmrPipe  -fn PS -p0 314.0 -p1 0.0 -di                    \
| nmrPipe  -fn EXT -x1 5.5ppm -xn 11.5ppm -sw \
| nmrPipe  -fn TP                                              \
| nmrPipe  -fn EXT -x1 1 -xn 18 -sw                    \
| nmrPipe  -fn SP -off 0.5 -end 1.0  \
| nmrPipe  -fn ZF  -size 320                               \
| nmrPipe  -fn FT -alt                                       \
| nmrPipe  -fn PS -p0 0.0 -p1 0.0 -di                     \
| pipe2xyz -out xy1/test%03d.ft2 -y

xyz2pipe -in fid2/test%03d.fid -x -verb                  \
| nmrPipe  -fn COADD -axis Y -cList 1 1 -1 -1 1 1 -1 -1 1 1 -1 -1 1 1 -1 -1 -time  \
| nmrPipe  -fn SP -off 0.5 -end 1.0  \
| nmrPipe  -fn ZF -size 32000                                \
| nmrPipe  -fn FT                                              \
| nmrPipe  -fn PS -p0 314.0 -p1 0.0 -di                    \
| nmrPipe  -fn EXT -x1 5.5ppm -xn 11.5ppm -sw \
| nmrPipe  -fn TP                                              \
| nmrPipe  -fn EXT -x1 19 -xn 36 -sw                    \
| nmrPipe  -fn SP -off 0.5 -end 1.0  \
| nmrPipe  -fn ZF  -size 320                               \
| nmrPipe  -fn FT -alt                                       \
| nmrPipe  -fn PS -p0 0.0 -p1 0.0 -di                     \
| pipe2xyz -out xy2/test%03d.ft2 -y

addNMR -in1 xy1/test%03d.ft2 -in2 xy2/test%03d.ft2 -out  F3F1-t2/test%03d.ft2 –add # sum of two 15N t1 data sets
```

```
xyz2pipe -in F3F1-t2/test%03d.ft2 -z -verb                    \
| nmrPipe  -fn SP -off 0.5 -end 1.0  \
| nmrPipe  -fn ZF -size 320                           \
| nmrPipe  -fn FT -alt -neg                               \
| nmrPipe  -fn PS -p0 0.0 -p1 0.0 -di               \
| pipe2xyz -out F3F2-F1/test%03d.ft3 -z

xyz2pipe -in F3F2-F1/test%03d.ft3 -x > hNcacoNH.ft
pipe2ucsf hNcacoNH.ft hNcacoNH.ucsf
```

References:

[1] H. L. Schmidt, L. J. Sperling, Y. G. Gao, B. J. Wylie, J. M. Boettcher, S. R. Wilson, C. M. Rienstra, *J Phys Chem B* **2007**, *111*, 14362-14369.

[2] a) B. H. Meier, *Chem Phys Lett* **1992**, *188*, 201-207; b) R. Verel, M. Baldus, M. Nijman, J. W. M. van Os, B. H. Meier, *Chem Phys Lett* **1997**, *280*, 31-39; c) M. Baldus, A. T. Petkova, J. Herzfeld, R. G. Griffin, *Molecular Physics* **1998**, *95*, 1197-1207; d) S. R. Hartmann, E. L. Hahn, *Physical Review* **1962**, *128*, 2042-2053.

[3] J. Cavanagh, W. J. Fairbrother, A. G. Palmer, M. Rance, N. J. Skelton, *Protein Nmr Spectroscopy: Principles and Practice, 2nd Edition* **2007**, 1-888.

[4] T. Gopinath, G. Veglia, *Angew Chem Int Ed Engl* **2012**, *51*, 2731-2735.

[5] P. Fricke, V. Chevelkov, M. Zinke, K. Giller, S. Becker, A. Lange, *Nat Protoc* **2017**, *12*, 764-782.

[6] T. Gopinath, G. Veglia, *J Magn Reson* **2019**, *310*, 106664.

[7] A. E. Bennett, C. M. Rienstra, J. M. Griffiths, W. G. Zhen, P. T. Lansbury, R. G. Griffin, *J Chem Phys* **1998**, *108*, 9463-9479.

[8] C. Shi, C. Oster, C. Bohg, L. Li, S. Lange, V. Chevelkov, A. Lange, *J Am Chem Soc* **2019**, *141*, 17314-17321.

[9] a) F. Delaglio, S. Grzesiek, G. W. Vuiste, G. Zhu, J. Pfeifer, A. Bax, *J Biomol NMR* **1995**, *6*, 277-293; b) T. Gopinath, G. Veglia, *Methods Mol Biol* **2018**, *1688*, 37-53.

[10] a) T. Gopinath, A. Kumar, *J Magn Reson* **2006**, *183*, 259-268; b) T. Gopinath, G. Veglia, *J Chem Phys* **2013**, *138*, 184201.

[11] W. Lee, M. Tonelli, J. L. Markley, *Bioinformatics* **2015**, *31*, 1325-1327.

[12] W. Lee, A. Bahrami, H. T. Dashti, H. R. Eghbalnia, M. Tonelli, W. M. Westler, J. L. Markley, *J Biomol NMR* **2019**, *73*, 213-222.

[13] L. B. Andreas, K. Jaudzems, J. Stanek, D. Lalli, A. Bertarello, T. Le Marchand, D. Cala-De Paepe, S. Kotelovica, I. Akopjana, B. Knott, S. Wegner, F. Engelke, A. Lesage, L. Emsley, K. Tars, T. Herrmann, G. Pintacuda, *Proc Natl Acad Sci U S A* **2016**, *113*, 9187-9192.

[14] a) V. S. Manu, A. Kumar, *Physical Review A* **2012**, *86*; b) V. S. Manu, G. Veglia, *J Magn Reson* **2016**, *273*, 40-46; c) G. Veglia, V. S. Manu, *United States Patent Application Publication* **2020**, *US 2020/0341084 A1*.

[15] N. C. Nielsen, H. Bildsoe, H. J. Jakobsen, M. H. Levitt, *J Chem Phys* **1994**, *101*, 1805-1812.

[16] T. Gopinath, G. Veglia, *J Magn Reson* **2015**, *253*, 143-153.

[17] T. Gopinath, G. Veglia, *J Magn Reson* **2012**, *220*, 79-84.

[18] a) T. Gopinath, D. K. Weber, G. Veglia, *J Biomol NMR* **2020**, *74*, 267-285; b) J. Stanek, T. Schubeis, P. Paluch, P. Guntert, L. B. Andreas, G. Pintacuda, *J Am Chem Soc* **2020**, *142*, 5793-5799.

[19] A. Wickramasinghe, S. L. Wang, I. Matsuda, Y. Nishiyama, T. Nemoto, Y. Endo, Y. Ishii, *Solid State Nucl Mag* **2015**, *72*, 9-16.

[20] A. C. Kolbert, A. Bielecki, *J Magn Reson Ser A* **1995**, *116*, 29-35.

[21] T. Westfeld, R. Verel, M. Ernst, A. Bockmann, B. H. Meier, *J Biomol NMR* **2012**, *53*, 103-112.

[22] K. O. Tan, A. B. Nielsen, B. H. Meier, M. Ernst, *J Phys Chem Lett* **2014**, *5*, 3366-3372.

[23] E. Barbet-Massin, A. J. Pell, K. Jaudzems, W. T. Franks, J. S. Retel, S. Kotelovica, I. Akopjana, K. Tars, L. Emsley, H. Oschkinat, A. Lesage, G. Pintacuda, *J Biomol NMR* **2013**, *56*, 379-386.

[24] D. H. Zhou, G. Shah, M. Cormos, C. Mullen, D. Sandoz, C. M. Rienstra, *J Am Chem Soc* **2007**, *129*, 11791-11801.
